# Ultraviolet-induced RNA:DNA hybrids interfere with chromosomal DNA synthesis

**DOI:** 10.1093/nar/gkab147

**Published:** 2021-03-10

**Authors:** Elena A Kouzminova, Andrei Kuzminov

**Affiliations:** Department of Microbiology, University of Illinois at Urbana-Champaign, Urbana, IL 61801, USA; Department of Microbiology, University of Illinois at Urbana-Champaign, Urbana, IL 61801, USA

## Abstract

Ultraviolet (UV) induces pyrimidine dimers (PDs) in DNA and replication-dependent fragmentation in chromosomes. The *rnhAB* mutants in *Escherichia coli*, accumulating R-loops and single DNA-rNs, are generally resistant to DNA damage, but are surprisingly UV-sensitive, even though they remove PDs normally, suggesting irreparable chromosome lesions. We show here that the RNase H defect does not cause additional chromosome fragmentation after UV, but inhibits DNA synthesis after replication restart. Genetic analysis implies formation of R-loop-anchored transcription elongation complexes (R-loop-aTECs) in UV-irradiated *rnhAB* mutants, predicting that their chromosomal DNA will accumulate: (i) RNA:DNA hybrids; (ii) a few slow-to-remove PDs. We confirm both features and also find that both, surprisingly, depend on replication restart. Finally, enriching for the UV-induced RNA:DNA hybrids in the *rnhAB uvrA* mutants also co-enriches for PDs, showing their co-residence in the same structures. We propose that PD-triggered R-loop-aTECs block head-on replication in RNase H-deficient mutants.

## INTRODUCTION

Hard ultraviolet (UVC, henceforth ‘UV’) is perhaps the best-studied DNA-damaging treatment, with the spectrum of UV-induced DNA lesions predominantly comprising cyclobutane pyrimidine dimers and 6-4 photoproducts (1). In UV-irradiated *Escherichia coli* cells incubated in the dark to avoid photoreactivation, both types of pyrimidine dimers (PDs) are exclusively removed by the UvrABC-promoted nucleotide excision-repair (NER) (2). Functionally, NER is further subdivided into global genomic repair (GGR, operating anywhere in the genome) and transcription-coupled repair (TCR, preferentially removing lesions from actively-transcribed genes) (3). TCR is triggered by RNA polymerase stalling at a PD in the template strand and is initiated by Mfd translocase, which displaces RNA polymerase and recruits NER enzymes to remove the transcription-stalling PD (4,5).

Rapid removal of UV-induced PDs from DNA by NER is even more important for DNA replication, since after sublethal UV exposure of growing *E. coli*, DNA synthesis cannot restart for about 20 min (6,7), while transcription and translation are barely affected (8). The consequences of replication forks encounters with PDs and with excision intermediates of PD removal are much more serious and lead to formation of either blocked single-strand gaps (so-called ‘daughter-strand gaps’) (9,10) or disintegrated replication forks (10,11). In *E. coli*, blocked ss-gaps are either closed by the RecFOR recombinational repair pathway, or by its backup via translesion DNA synthesis, mostly by Pol V across PDs, but also by Pol IV (10). Disintegrated replication forks are reassembled by the RecBC-pathway of recombinational repair (10); its absence in the *recBC* mutants reveals that UV induces high levels of chromosome fragmentation (12).

**Figure 1.**
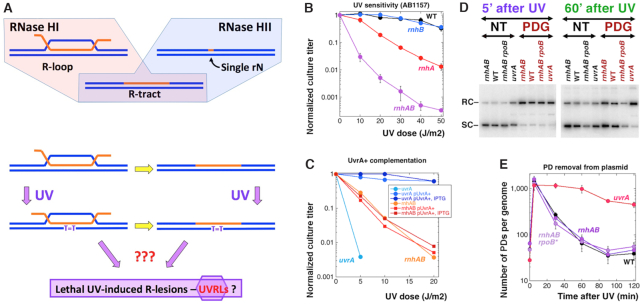
The *rnhAB* double mutants are strongly sensitive to UV, but are not defective in NER. (**A**) Top, substrates of the RNase HI and RNase HII enzymes in *E. coli*. Bottom, R-loops are speculated to occasionally transform into R-tracts, and both could be further exacerbated by UV-lesions (T=T), yielding mysterious lethal UV-induced R-lesions (UVRLs). (**B**) UV-irradiation survival. From here on, all reported values are means of at least three independent measurements ± SEM. Assume no significant difference when error bars are almost touching. When error bars are invisible, they are covered by the symbols. The strains for panels B–E are: WT, AB1157; Δ*rnhA*, L-413; Δ*rnhB*, L-415; Δ*rnhAB*, L-416; *uvrA*, SRK303; rnhAB rpoB*35, L-416-33. (**C**) The effect on UV-irradiation survival of UvrA^+^ overproduction. The *uvrA* mutant is shown as a control for UvrA production from the plasmid pSRK10-1. (**D**) Plasmid relaxation by PD-glycosylase as a generic PD detection assay. A representative gel of only two time points is shown. NT, no treatment; PDG, PD-glycosylase treatment; RC, relaxed circular plasmid; SC, supercoiled plasmid. The plasmid is pEAK86. (**E**) Number of PDs per genome, as quantified from gels like in ‘D’. Note that the background of the plasmid relaxation procedure (the 0 min point is taken before UV) is ∼45.

Since everything seems to be known about how UV damages DNA and what chromosomal consequences of this damage are, at least in *E. coli*,—it comes as a surprise to find a strong UV-sensitivity in mutants that have all the known UV-repair pathways intact, as this suggests existence of yet-to-be-characterized UV-induced chromosome lesions. We have serendipitously found an unexpectedly strong UV-sensitivity in the *rnhA rnhB* double mutant of *E. coli* (henceforth *rnhAB*), deficient in both RNase H enzymes, ribonucleases attacking the RNA moiety of the RNA:DNA hybrids (RDHs). The RNase H-deficient mutants accumulate single rNs and R-loops in their genomic DNA, with dramatic consequences. RNase H-deficient mutants are inviable in higher eukaryotes (13,14) and show various defects in lower eukaryotes (15,16) and bacteria (17,18). *Escherichia coli* has two RNase H enzymes, with distinct specificities (Figure 1A, top). RNase HI encoded by the *rnhA* gene, removes R-loops and ≥4 nt rN-runs embedded in DNA (R-tracts) (19,20). RNase HII, encoded by the *rnhB* gene, also called the ‘junction ribonuclease’, incises the 5′RNA-DNA3′ junction within dsDNA, leaving a single rN on the 5′ cleaved end and thus initiating removal of single DNA-rNs, and also R-tracts (Figure 1A) (21,22).

Unlike analogous eukaryotic RNase H2 enzymes, the prokaryotic RNase HII shows no activity against RDHs lacking RNA–DNA junctions (unless metallated with Mn^2+^ instead of Mg^2+^) (23,24). Therefore, in contrast to the higher eukaryotes, *E. coli rnhB* mutants, although accumulating measurable density of single rNs in the genome, show no growth defects or other gross phenotypes, indicating that single rNs at this density do not interfere with DNA replication (18). In contrast, bacteria deficient in RNase HI (*rnhA* mutants in *E. coli*) grow slower and suffer from unscheduled initiation of the chromosome replication and synthetic lethality in combination with various defects in the DNA metabolism (25–27). This implicates RNA:DNA hybrids in general and (protein-free) R-loops in particular as impediments to replication,—an idea especially popular in eukaryotes (28,29). However, protein-free R-loops are rather unstable and should disassemble when a replication fork approaches with the accompanying wave of positive supercoiling; in contrast, R-loops associated with transcribing RNA-polymerases are argued to represent a considerable challenge (30). The lethality of unresolved conflict of replication with head-on transcription was recently demonstrated (31,32) and was proposed to be due to formation of R-loop-anchored transcription elongation complexes (R-loop-aTECs), which can be disassembled only by RNase H enzymes, becoming irreparable chromosome lesions in their absence (30).

The relatively modest phenotypes of the *rnhA* single mutants in *E. coli* are remarkably exacerbated by the *rnhB* defect: the resulting completely RNase H-deficient *rnhAB* double mutants grow extremely slowly, produce filamentous cells, are highly induced for SOS-response and, because of high levels of chromosome fragmentation, depend on recombinational repair (18). To explain severe chromosomal problems of the *rnhAB* double mutants, we argued that R-loops in them are converted into a common substrate for both RNase HI and RNase HII,—the so-called R-tracts (Figure 1A, top), which are then transformed, via R-gaps, into double-strand breaks (18). However, we could not detect the expected R-tracts in plasmid DNA from *rnhAB* mutants,—and so we sought conditions that would kill the *rnhAB* mutants, reasoning that under these lethal conditions, the elusive R-tracts could be amplified and become detectable.

In some *E. coli* strains expressing a ‘steric gate’ mutant of the translesion DNA polymerase Pol V with the increased capacity for DNA–ribonucleotide incorporation, NER was implicated in removal of single DNA–rNs (33). Moreover, the PD-sensor of NER, the UvrA protein, was also shown to recognize rNs in dsDNA substrates in vitro (33), although this finding was later disputed (34). In support of the idea that NER removes misincorporated ribonucleotides in the *rnhAB* mutants, we did observe an additional growth defect in the *rnhAB uvrA* triple mutant, although the density of single DNA–rNs in this mutant remains the same as in its *rnhAB* (UvrA+) parent (18). It is while verifying the extreme UV-sensitivity of the *uvrA* mutants, that we unexpectedly found the high UV sensitivity of the *rnhAB* (NER+) mutants (Figure 1B and Supplementary Figure S1A).

Our initial reasoning about the UV sensitivity of *rnhAB* NER+ mutant envisaged a combination of pre-existing R-loops or R-tracts with induced PDs to produce lethal ‘UV-induced R-lesions’ (UVRLs) (Figure 1A, bottom). However, after testing various models of UVRL formation, we conclude that UVRLs represent R-loop-aTECs, formed as a result of transcribing RNA polymerases stalling at PDs, which are subsequently aggravated by restart of DNA synthesis.

## MATERIALS AND METHODS

### Bacterial strains

*Escherichia coli* strains (all K-12) are described in Supplementary Table S1, while plasmids are described in Supplementary Table S2. Strain construction was by P1 transduction (35) or by deletion-replacement method with the following removal of the antibiotic resistance by pCP20 (36). Deletions-replacements were confirmed by PCR. The *recA*, *recBCD*, *recF* and *uvrA* mutants were confirmed by their characteristic UV-sensitivities, while the *dnaC* mutants were verified as unable to grow at 42°C.

**Primers** used for making deletion-replacements, PCR amplification, sequencing and verification of indicated chromosomal loci are listed in the Supplementary Table S3 (Primers).

### Media and growth conditions

Cells were grown in LB broth [10 g tryptone, 5 g yeast extract, 5 g NaCl per liter (pH 7.2), with NaOH] or on LB plates (15 g agar per liter of LB broth). The growth temperature was 28°C unless otherwise indicated in the description of experiments. When screening for mutations linked to antibiotic-resistant genes or when the cells were carrying plasmids, the media were supplemented with the required antibiotic: 100 μg/ml ampicillin, 50 μg/ml kanamycin, 10 μg/ml tetracycline or 10 μg/ml chloramphenicol. 1 mM IPTG was used for UvrA overproduction from pSRK10-1.

### Viability tests

Overnight cultures of the tested strains were diluted 100-fold in the morning and grown in fresh LB to OD_600_ ∼0.2, after which the treatment was delivered.

#### UV treatment and survival

Five 10-fold serial dilutions of six cultures were made in sterile 1% NaCl solution and spotted by 10 μl in one row for each dilution on several square Petri dishes with LB agar. Spots were dried, and the plates were exposed to UVC-light (254 nm) in Hoefer UVC 500 UV cross-linker. All manipulations were performed under yellow light (F15T8-GO lamp, General Electric) to avoid photoreactivation. The time to deliver a particular UV dose was calculated from the measurements of UV irradiation with UVC Digital Light Meter (model UV512C, General). Plates were developed overnight in the dark at 28°C, and the still pin-prick colonies were counted under a stereomicroscope. The survival was determined as the ratio of a culture titer after a particular UV exposure to the titer of the untreated culture.

#### Rifampicin pretreatment

One half of the culture was treated with 100 μg/ml of rifampicin for 5 min, the other half was left untreated. To remove rifampicin, cells were pelleted and resuspended in fresh LB twice, followed by UV exposure of the culture serial dilutions on LB plates.

#### DNA damaging treatments

A specific amount of the agent was added directly to exponentially growing cultures at 28°C. The treatments were: 10 mM MMS for 30 min, 10 μg/ml mitomycin C for 30 min, 10 mM hydrogen peroxide for 15 min, 30 μg/ml nalidixic acid at 37°C (two time points). Aliquots of cultures were taken, serially diluted in 1% NaCl and spotted on LB agar to enumerate survivors. The plates were incubated overnight at 28°C. The survival was determined as the ratio of the colony forming units at a specific treatment time or agent concentration to the colony forming units before the treatment (zero time) or without the agent.

### Processing cultures to determine the density of DNA–ribonucleotides, thymine dimers or RNA/DNA hybrids (RDHs)

Strains, including those harboring appropriate plasmids, were grown in 20 ml of LB to OD_600_ of 0.4, collected by centrifugation, resuspended in 4 ml of 1% NaCl. 1 ml of cells was taken to purify DNA as no UV control, the other 3 ml were mixed with 12 ml of 1% NaCl and transferred to an open sterile glass tray (14 cm × 23 cm), placed under the GE germicidal lamp emitting 254-nm UV-light, and exposed to UV-light with slow shaking of the tray on a platform. After irradiation, the cells were diluted (1:1) with 2× LB without NaCl and transferred to a sterile flask for post-irradiation recovery at 37°C with vigorous shaking. Seven-milliliter samples were removed at specific times, placed on ice, then collected by centrifugation at 7000 rpm for 5 min and processed according to the ‘total genomic DNA isolation’ protocol. Samples designated for PD density analysis by plasmid nicking were first mixed with four volumes of ice-cold 0.01M KCN, 10% pyridine stop solution (37) and processed according to the plasmid isolation protocol.

#### Plasmid DNA isolation

Plasmid DNA was extracted according to the small-scale alkaline lysis plasmid isolation protocol with all steps carried out on ice (18) followed by LiCl purification step (38).

#### ‘Total genomic ‘ DNA isolation

Cell lysates were prepared according to Brij lysis procedure (39). This method was crucial to obtain highly reproducible results with the S9.6 antibodies. In particular: after UV exposure and post-irradiation recovery (described above), cells were pelleted and resuspended in 0.25 ml of ice-cold solution of 30% sucrose, 0.05M Tris–HCl, pH 8.0. Freshly prepared lysozyme, 0.05 ml of 5 mg/ml in 0.25 M Tris–HCl, pH8.0, was added to the cell suspension, which was kept on ice for 5 min with gentle mixing. 0.1 ml of 0.25 M EDTA pH 8.0 was added for another 5 min on ice with occasional gentle swirling. To lyse the cells, 0.4 ml of detergent mixture (1% Brij58, 0.4% sodium deoxycholate, 0.0625 M EDTA and 0.05M Tris–HCl, pH 8.0) and RNase A to the final concentration 20 μg/ml were added and gently mixed. The samples were kept on ice for 30–60 min followed by three organic extractions: 0.8 ml phenol with 40 μl chloroform, followed by 0.8 ml phenol/chloroform (1:1), followed by 0.8 ml chloroform. The final aqueous phase was transferred into fresh tube and precipitated with salt and ethanol.

DNA concentrations were measured with Qubit 2.0 Fluorometer (Invitrogen).

### Quantification of ribonucleotides and PDs by the plasmid relaxation method

Density of ribonucleotides in DNA was calculated after plasmid DNA was treated with RNase HII (NEB) or RNase HI (Life Technologies) as previously described (18).

PD density was calculated in plasmid DNA treated with T4 endonuclease V (NEB). In particular, 100–200 ng of purified plasmid DNA was treated in 20 μl of 1× T4 PDG reaction buffer (25 mM sodium phosphate (pH 7.2), 100 mM NaCl, 1 mM EDTA, 1 mM dithiothreitol) containing either no enzyme or 3 units of T4 PDG (NEB) at 37°C for 30 min. After enzymatic reactions the plasmid intermediates were analyzed in 1% agarose gels followed by Southern hybridization with a radioactive probe made from the appropriate plasmid DNA by random priming (18). The signals from supercoiled and relaxed plasmid species were quantified by Phosporimager (FujiFilm FLA-3000, Fuji).

Average density of nicks in a plasmid DNA was derived from the remaining supercoiled DNA representing zero class of the Poisson distribution after the enzymatic treatment. Our calculations steps were (40): (i) the radioactivity signal in the supercoiled monomer band was divided by the sum of the radioactivity in the supercoiled and relaxed monomer bands in the lane to determine the fraction of the supercoiled species, (ii) average number of nicks was calculated according to the formula –ln(*F*_treated_/*F*_untreated_), where *F*_treated_ is a fraction of supercoiled band from the lane with the enzyme treatment, *F*_untreated_ is a fraction of supercoiled band from the lane of the plasmid treated with buffer-only to take into account the background nicking. The density of nicks in a plasmid was calculated by dividing the double-stranded-plasmid length (in nucleotides) per average number of nicks; then the *E. coli* genome (9.2 × 10^6^ nt) was divided by the density of nicks to get the number of PDs per genome.

### Quantification of RDHs and PDs in the chromosomal DNA

Cells were grown and UV-irradiated as described above. DNA was extracted by the total genomic DNA isolation method.

For RDH signal detection: 500 ng of the DNA was split in half and run on two gels (0.8% agarose in TAE buffer) under identical conditions. One gel was stained with ethidium bromide, bands were visualized and then the gel was treated for Southern analysis as before (18). The second gel was soaked in 0.5× TBE buffer, and DNA was transferred onto Amersham Hybond-N^+^ membrane (GE Healthcare) by electric transfer using a Trans-blot cell (Bio-Rad) for 16 h in 0.5× TBE buffer, 20 V with cooling. After electric transfer, the DNA was UV-crosslinked to the membrane, and standard western blotting procedure was applied. Briefly, blocking of the membrane was done for 1 h at room temperature with rotary platform shaker with 5% non-fat milk dissolved in TTBS buffer (0.02 M Tris–HCl, pH 7.5, 0.9% NaCl, 0.05% v/v Tween 20), then the solution was exchanged for the buffer with the primary antibodies (Anti-DNA–RNA Hybrid, clone S9.6 monoclonal antibodies (Millipore)) diluted 1:5000 in TTBS, 5% milk buffer and the membrane was probed at room temperature for 1 h, followed by three washes 10 min each with TTBS buffer. Secondary antibodies (Anti-Mouse IgG-peroxidase antibody, Sigma-Aldrich) were diluted to 1:10 000 in TTBS, 5% milk buffer and applied to the membrane at room temperature for 1 h, followed by three washes 10 min each with TTBS buffer. Signal was developed with Supersignal West Femto Maximum Sensitivity Substrate kit (Thermo Scientific). Imaging was done with CCD camera of the Bio-RAD ChemiDoc XRS system and Bio-Rad Quantity One software. Image analysis and quantification of the TIFF files was done with ImageQuant TL program (GE HealthCare Life Sciences).

For thymine dimer signal detection, the same procedure as for the RDH detection was followed, with some modifications. Briefly, 500 ng/lane genomic DNA samples were analyzed by gel electrophoresis. The gels with the DNA samples prepared to be analyzed by Western blotting were treated as for the Southern analysis, since the Anti-Thymine Dimer antibodies (KTM53, Kamiya Biomedical MC-062) bind single stranded DNA. Vacuum transfer of the DNA to the Amersham Hybond-N^+^ membrane (GE Healthcare) was carried out, the membrane was baked for 2 h at 80°C (instead of UV-crosslinking) and probed with Anti-Thymine Dimer antibodies diluted 1:5000 as primary antibodies.

### Calculations

In each experiment, Western signal of a particular strain was divided by the corresponding Southern genomic DNA signal to obtain either RDH density or PD density value. The RDH density for each strain at a particular post-UV time point was normalized to the RDH density value of the *rnhAB* log culture within the same experiment. Similarly, the PD density value for each post-UV time point was normalized to the PD density value in the same strain measured at 5 min post-UV (which was taken for 100%).

### Measurement of the DNA synthesis rate

Cells from 2 ml log cultures grown at 30°C to OD_600_ = 0.35 were centrifuged, washed once with 2 ml 1% NaCl and resuspended in 1.5 ml 1% NaCl, 0.01% Triton X100 to yield OD_600_ = 0.4. For zero time point: 300 μl of the sample was mixed with 300 μl of 2× LB without NaCl and the rate of DNA synthesis was measured as below. The rest of the sample (1.2 ml) was transferred to a sterile Petri plate and spread in a thin layer. UV irradiation was performed with Hoefer UVC 500 UV cross-linker. Following irradiation, 1 ml was transferred to a tube with 1 ml of 2× LB without NaCl. Tubes were shaken for up to 3 h at 37°C in the dark, and 200 μl aliquots were removed for analysis at the indicated times.

To measure the rate, 200 μl of the sample was mixed with prewarmed at 37°C 200 μl LB containing 1 μCi of [methyl-^3^H] thymidine (MP Biomedicals) and 0.4 μg of thymine. The reaction was carried out for 3 min and then stopped by addition of 5 ml of ice-cold 5% TCA. The tubes were kept on ice throughout the experiment. Samples were processed as described (12). During post-irradiation recovery, cultures were diluted 2-fold at 50, 90 and 150 min, if needed to keep cultures in logarithmic growth. Dilutions were taken into account after the amount of ^3^H was determined by scintillation counting.

### Chromosome fragmentation by pulsed-field gels

Overnight cultures were diluted 100-fold in LB supplemented with 5 μCi/ml of ^32^P-orthophosphoric acid and grown to the OD_600_ of 0.35 at 28°C. At this point, the medium was changed to 1% NaCl, 0.01% Triton X100 for UV irradiation in Petri plates, as described above for the procedure to measure DNA synthesis rate. Following irradiation, 1 ml was transferred to a tube with 1 ml of 2× LB without NaCl supplemented with the ^32^P-orthophosphoric acid and shaken at 37°C for 2 h in the dark. No-UV control samples were treated the same way, but after 1 h recovery time at 37°C the cultures were diluted 4-fold in fresh LB with the ^32^P-orthophosphoric acid, to keep them growing exponentially. The chromosomal DNA preparation in agarose plugs, treatments and conditions for pulse-field gel electrophoresis, as well as quantification of the chromosomal breakage were done as before (41).

### RNase sensitivity tests of the RDH

Treatments of 0.5 μg of the total genomic DNA with RNase HI (Takara) or RNaseHII (NEB) were performed as described (18). It was critical to run the reaction samples on the gel followed up by electric transfer to separate RNase HI from the substrate. The enzyme tightly binds to the substrate (even at 0°C in water) and completely blocks immunodetection if a reaction mix is directly used in the dot-blot procedure.

#### RNaseA treatment

Treatment of 0.5 μg of total genomic DNA was in 20 μl of the reaction buffer [20 mM Tris–HCl (pH 8.0), 40 mM KCl, 8 mM MgCl_2_, 1 mM dithiothreitol] containing either no enzyme or 5 μg/ml of RNase A at 37°C for 15 min. If inhibition of RNaseA activity toward dsRNA was desired, the reaction mixture was made 0.5 M for NaCl.

### DNA/RNA hybrid immunoprecipitation assay (DRIP)

Growth of the cultures and UV-irradiation were performed as described above in the section ‘Processing cultures to determine ribonucleotide density, thymine dimer density and RNA/DNA hybrids after UV irradiation’. Total nucleic acids were purified as described in the section: ‘Total genomic DNA isolation’.

For each DRIP experiment, 8–10 μg of DNA were digested with various enzymes: BamHI + EcoRI, or HaeII, or HaeIII in the 1xCutSmart Buffer (NEB) at 37°C, followed by extractions with phenol, phenol/chloroform, chloroform and ethanol precipitation.

After digestion with the restriction enzymes, the control DNA samples were treated with RNase HI (Takara). To verify completion of digestion and RNase HI treatment, 0.3–0.5 μg of the digested samples were run on 0.7% agarose gel.

The DRIP procedure was as described (31) with small modifications. The DRIP mixture was assembled in 0.4 ml volume by mixing 0.04 ml of 10× IP Binding buffer (200 mM Tris pH 7.5, 9% (1.55 M) NaCl, 0.5% Tween 20) and DNA sample in TE (10 mM Tris–HCl, pH 8.0, 1 mM EDTA) with 1/20 of the DNA sample removed beforehand to use as the INPUT control). S9.6 monoclonal antibodies (Millipore) were added (1 μl antibodies per 1 μg DNA) to the DRIP mixture and incubated at 4°C overnight with gentle rotation. Next morning, Protein A-Sepharose beads (BioVision) were prepared by washing them in 1× IP binding buffer (0.5 ml of buffer per 0.1 ml of the beads slurry) three times for 5 min each with gentle rotation at room temperature and precipitation at 2000 rpm for 1 min. 0.04 ml of the prepared ProteinA-Sepharose beads were added to 0.4 ml of DRIP mixture, and the suspension was incubated for 4 h at 4°C with gentle rotation. The DRIP-beads mixture was pelleted at 2000 rpm for 1 min. The supernatant was removed, 0.1 ml was twice ethanol/salt precipitated, resuspended in 0.12 ml CHIP/Proteinase buffer [10 mM Tris pH 8.0, 1 mM EDTA, 0.67% SDS, 420 μg/ml Proteinase K (Roshe)] and processed in parallel to the DRIP fractions (below) to generate the S9.6-DNA-unbound (Eluate) fraction. The pelleted DRIP-beads (the DRIP fraction) were washed in 1 ml of 1× IP Binding Buffer three times with the additional wash in 1 ml TE buffer. After washes, 0.12 ml of the CHIP/Proteinase buffer were added to the DRIP-beads and incubated at 55°C for 45 min with shaking the tubes in a Eppendorf Thermomixer at 800 rpm. Beads were collected by centrifugation at 7000 rpm for 1 min, and the supernatant (the DRIP fraction) was transferred to a new tube. The DRIP and Eluate DNA was phenol-chloroform extracted, mixed with glycogen (Thermo Scientific) at 0.2 μg/μl final concentration, made 0.5 M for NaCl, 2.5 volumes of ethanol were added, and after mixing incubated for 2–18 h at −20°C, then centrifuged at room temperature for 30 min at 16 000 × g. DNA pellets were dissolved in 30 μl TE buffer and analyzed by dot blotting.

All DNA samples were split into two fractions: 5 μl for RDH signal and 25 μl for PD and DNA signal analysis. For RDH western analysis, 5 μl of DNA after being mixed with 45 μl of TE buffer, were applied directly to the membrane (Hybond N+, GE). For PD and DNA blotting, 25 μl sample was denatured for 15 min in 0.1M NaOH at 37°C, then neutralized with 0.2 M Tris–HCl pH 8.0, followed by addition of TE (25 μl DNA, 25 μl 0.2 M NaOH, 50 μl 0.4 M Tris–HCl, 100 μl TE). All DNA samples were applied to the membrane using Hybrid-Dot Manifold (Life Technologies, Inc). For RDH signal analysis, the membrane was UV-crosslinked, while the membrane for PD and DNA signal determination was baked overnight at 80°C. Detection of the RDH and PD signals was done by western analysis with the corresponding antibodies, as described above in ‘Quantification of the RDHs and PDs in the chromosomal DNA’.

After western analysis, the membrane with PD signal was washed off for 30 min at 37°C with 2% SDS in 50 mM Tris–HCl, and then the standard Southern hybridization protocol was performed with the total genomic DNA as the radioactive probe (generated by random priming). DNA images were generated with phosphor-imaging screens using Typhoon FLA 7000 (GE Healthcare) and calculated with ImageQuant TL program (GE HealthCare Life Sciences).

#### Enrichment calculations

RDH densities in three fractions (Input, DRIP, Eluate) were calculated by dividing the Western RDH signals to the corresponding Southern genomic DNA signals. RDH-enrichment was calculated as the ratio of the RDH densities in the DRIP or Eluate fractions to the RDH density of the corresponding INPUT fraction. The same procedure was applied to calculations of the PD-enrichment from PD densities.

### Co-enrichment of RDHs and PDs

Cells growth, UV-irradiation and DNA extraction was done as described in ‘Quantification of RDHs and PDs in the chromosomal DNA’. 10 μg of DNA was digested with *Hae*II in 200 μl reaction in the 1xCutSmart Buffer (NEB) at 37°C, followed by extractions with phenol, phenol/chloroform, chloroform and ethanol precipitation. DNA samples were split in half and run on two agarose gels. Treatment of the gels, processing of the membranes and western analysis were as described in ‘Quantification of RDHs and PDs in the chromosomal DNA’, the only difference being that DNA from both gels was transferred to the membranes by electric transfer. Western signal imaging was done with iBright CL1000 Imaging System (Invitrogen). Image analysis and quantification of the TIFF files was done with ImageQuant TL program (GE HealthCare Life Sciences).

#### Calculation of co-enrichment profiles

The profile of the lane is divided into 16 equal fractions from the well to the 0.2–0.5 kb bottom of the lane. The density values for RDH and PD are calculated for each fraction via dividing western signal by Southern (DNA) signal. The RDH density for 30 min point is then normalized to the density of RDH from the corresponding fractions of the log sample (normalized RDH density = RDH 30 min density/RDH log density). Similarly, the PD density of the 30 min point is normalized to the density of PD from the 5 min post-UV sample (normalized PD density = 30 min PD density/5 min PD density). This normalization step permits meaningful comparison of data points from different experiments. The co-enrichment value for a particular fraction within the lane is calculated by multiplying the normalized PD and RDH densities of this fraction.

## RESULTS

### UV-sensitivity of *rnhAB* mutants is not due to defects in global NER or recombinational repair

Since it was proposed that UvrA excises single DNA-rNs in the *rnhAB* mutants (33,42), the UV-sensitivity of the *rnhAB* mutants (Figure 1B and Supplementary Figure S1A) could be due to UvrA protein titration by these DNA-rNs, making *rnhAB* mutants partial *uvrA* phenocopies. Yet, overexpression of the *uvrA*+ gene from a plasmid, that complemented UV-sensitivity of the *uvrA* control strain, failed to improve UV sensitivity of the *rnhAB* mutant (Figure 1C). We also assessed the global NER capacity of the *rnhAB* mutant by isolating plasmid DNA from cells irradiated with 36 J/m^2^ UV and treating it *in vitro* with T4 Endonuclease V (T4 PD glycosylase), that has also AP-lyase activity (43), nicking DNA at PD sites and thus relaxing supercoiled plasmid (Figure 1D), facilitating determination of the density of PDs in the plasmid DNA. At 5 min post-UV, this density translates into ∼1500 PDs per *E. coli* genome (∼42 PD per 1 J/m^2^) (Figure 1E). In the *uvrA* mutant, we observed no PD removal within the first hour, confirming its NER-deficiency (the 2 h partial PD ‘disappearance’ likely reflects replication of PD-free plasmid). In contrast, in all NER-proficient strains independently of their *rnhAB* status, we observed the same rate of PD removal from the plasmid DNA: about half of PDs was removed by 20 min, and almost all of them were removed by 60 min after UV (Figure 1E), demonstrating normal global NER capacity in the *rnhAB* mutants.

Since rapidly growing *rnhAB* double mutants form DSBs, induce substantial SOS response and rely on recombinational repair to reassemble fragmented chromosome (18), *rnhAB* mutants could be sensitive to UV because of an overwhelmed recombinational repair. If so, the *rnhAB* mutants should be also sensitive to all kinds of DNA damage, as recombinational repair mutants are (10). To test this possibility, we treated the *rnhAB* and *recBCD* mutants, along with the control strains, with various DNA-damaging agents. The *rnhAB* double mutant was indeed somewhat sensitive to DNA gyrase inhibitor nalidixic acid at 37°C (Supplementary Figure S1B). At the same time, the *rnhAB* mutant showed WT resistance against the DNA methylation agent methyl methanesulfonate (MMS), the DNA alkylating and crosslinking agent mitomycin C, and the oxidative agent hydrogen peroxide (while control mutants were killed by 2–6 orders of magnitude (Supplementary Figure S1C–E). Thus, RNase H-deficient *E. coli* mutants are not depleted for their global NER or recombinational repair capacities and therefore are generally DNA repair-proficient. We reasoned that some UV-induced PDs combined with RDHs accumulating in the RNase H-deficient mutants could create irreparable chromosome lesions (UVRLs, Figure 1A), — and set out to understand their nature.

### Translesion synthesis and the RecFOR pathway do not generate or repair UVRLs

**Figure 2.**
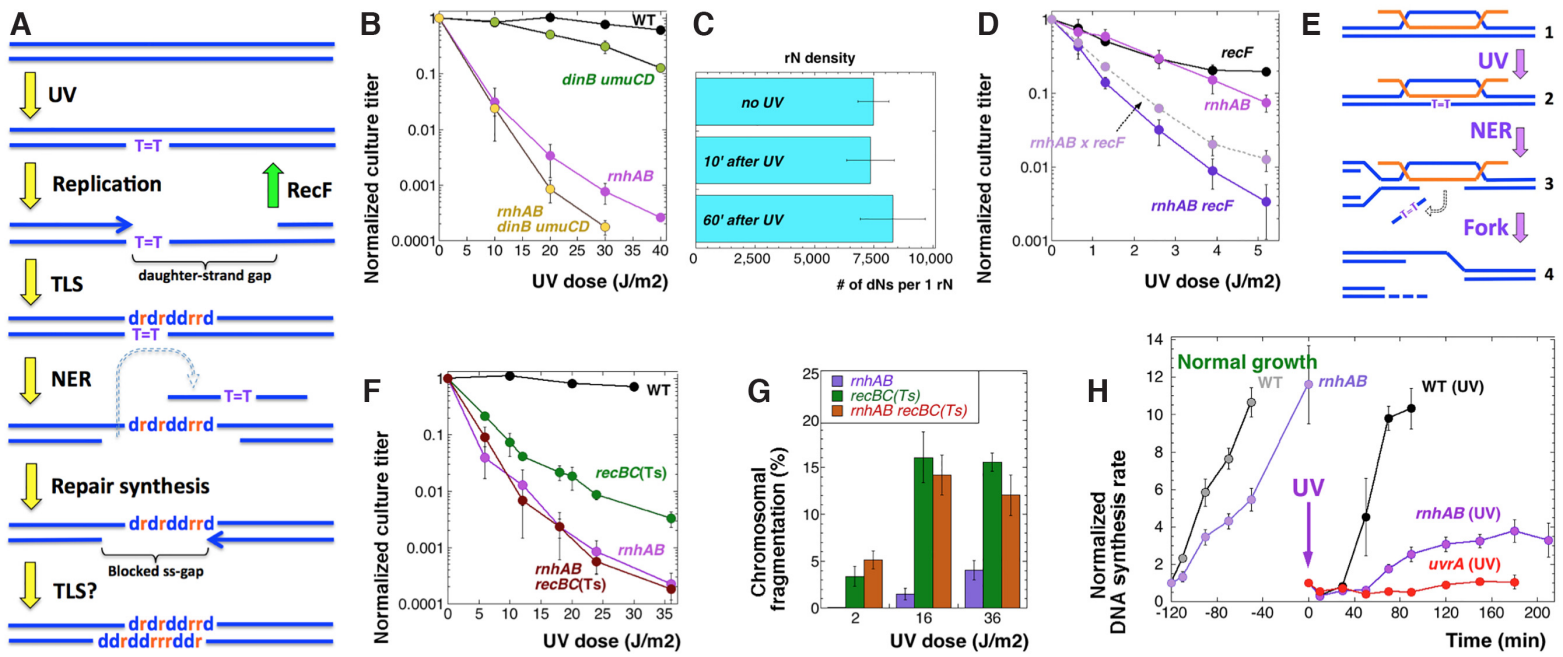
The UV-sensitivity of the *rnhAB* mutants is not due to translesion DNA synthesis, overwhelmed daughter-strand-gap repair or additional double-strand breaks. (**A**) A scheme explaining how translesion DNA synthesis could generate a two-strand R-lesion at PD. The full scheme is shown in Supplementary Figure S2. (**B**) Inactivation of the TLS polymerases fails to improve the UV-sensitivity of the *rnhAB* double mutant. The strains are: WT, AB1157; *dinB umuCD*, L-500; *rnhAB*, L-416; *rnhAB dinB umuCD*, L-497. (**C**) Density of ribonucleotides in DNA does not increase after 100 J/m^2^ UV. The DNA-rN density was determined by the plasmid relaxation with RNase HII *in vitro*. The data are combined averages of four different plasmids isolated from either *rnhAB* (L-416) or *rnhAB uvrA* (L-407) mutants (there was no difference). (**D**) The UV-sensitivity of the *recF*, *rnhAB* and *recF rnhAB* mutants. The dotted line represents predicted cumulative UV-sensitivity for the *recF rnhAB* strain. The strains are: *recF*, AM3; *rnhAB*, L-416; *recF rnhAB*, L-435. (**E**) Replication fork collapse at aborted PD excision opposite R-loop. If PD across an R-loop (2) is excised by NER (3), the resulting ssRNA gap cannot be closed by synthesis. Note that disintegrated replication forks are repaired by the RecBC pathway. (**F**) The *rnhAB* defect is epistatic over the *recBC*(Ts) defect for UV-sensitivity. The strains for panels F and G are: WT, AB1157, *recBC*(Ts), SK129; *rnhAB*, L-416; *recBC*(Ts) *rnhAB*, L-476. (**G**) UV-induced chromosomal fragmentation in the *recBC*(Ts), *rnhAB* and *recBC*(Ts) *rnhAB* mutants. (**H**) Recovery of DNA synthesis after 36 J/m^2^ of UV, administered at time 0. The DNA synthesis rates for unirradiated control cultures, measured at various times, were normalized to the values of the first measurement. The DNA synthesis rates for UV-treated cultures were normalized to the value just before the UV treatment (time 0). The strains are: WT, AB1157; *rnhAB*, L-416; *uvrA*, SRK303.

Due to high SOS-induction in the *rnhAB* mutants (18), the SOS-induced TLS polymerases could contribute to formation of the UVRLs by bypassing pyrimidine dimers with insertion of rNs instead of dNs (44), generating an R-tract or an R-patch (a short R-tract or a mixed run of rNs and dNs) (Figure 2A). In particular, Pol V polymerase is known to readily use rNTPs for translesion synthesis (45). If PD is then removed by NER from the template strand, and ss-gap filling is blocked by the R-patch, TLS could again fill the gap with another R-patch (Figure 2A and Supplementary Figure S2) creating a double stranded R-lesion, which in the RNase H-deficient strain could permanently block replisome passage.

This model predicts: (i) an improved UV-survival of the *rnhAB* mutants with additional mutations in Pol IV (*dinB*) and Pol V (*umuCD*) polymerases; (ii) a synergistic UV sensitivity of the *rnhAB recF* triple mutant, in which RecFOR-dependent recombinational repair of daughter-strand gaps is blocked (10) (Figure 2A and Supplementary Figure S2), leaving TLS as the only pathway to fill the ss-gaps to produce more R-patches. Contrary to these predictions, the *rnhAB umuCD dinB* quintuple mutant shows slightly elevated sensitivity at higher UV doses, which parallels the modest sensitivity of the *umuCD dinB* triple mutant itself at these doses (Figure 2B) (46) likely reflecting formation of lesions processed only by TLS. Further, we observed no increase in the density of ribonucleotides in plasmid DNA, isolated from growing or UV-irradiated *rnhAB* or *rnhAB uvrA* mutants, tested for presence of single rNMPs or R-patches in the plasmid relaxation assay (18) with either RNase HII (Figure 2C) or RNase HI (Supplementary Figure S3).

Finally, although the *rnhAB recF* mutant is more sensitive to UV than either *recF* or *rnhAB* mutants, there is no synergy between the *recF* and *rnhAB* defects, as UV-survival of the triple *rnhAB recF* strain is similar to the product of the two mutant survivals (Figure 2D), suggesting that handling of the UVRLs in the *rnhAB* mutants is not channeled through the RecF-dependent repair of blocked ss-gaps (therefore, UVRLs are not blocked ss-gaps). Thus, our genetic analysis suggests that TLS is not responsible for generating UVRLs in the *rnhAB* mutants, whereas the RecFOR repair of blocked ss-gaps neither mends UVRLs, nor participates in their formation.

### UVRLs are not resolved into double strand breaks in the *rnhAB* mutants

Even sublethal doses of UV cause significant chromosome fragmentation in *E. coli* via replication fork disintegration (12). The broken replication forks in wild type *E. coli* are robustly reassembled by the RecBCD-promoted recombinational repair (10), so the fragmented chromosomes are only detectable in the UV-sensitive *recBC* mutants, as linear DNA species in pulsed-field gels (12). If UVRLs in the *rnhAB* mutants cause replication fork disintegration, for example initiated by attempted repair of PD across R-loop (Figure 2E), then there will be synergistic decrease in UV-survival of the *rnhAB recBC* mutant, compared to both the *rnhAB* and *recBC* mutants, as well as *additional* fragmentation in UV-irradiated cultures of the *rnhAB recBC* mutant compared to the UV-induced chromosome fragmentation in the *recBC* mutant (12).

We showed before that the *rnhAB* mutant in AB1157 background is not temperature sensitive, but *rnhAB recBC*(Ts) mutants are synthetic lethal at 37°C (non-permissive temperature for *recBC*(Ts)) and accumulate DSBs after 4 h of growth at this temperature (18). To avoid this DSB formation and lethality, we incubated plates with UV-irradiated *rnhAB recBC*(Ts), *rnhAB* and *recBC*(Ts) cells at 38°C for only 2 h and shifted them to 28°C to allow colony formation. We detected no additional UV-sensitivity of the *rnhAB recBC* mutant compared with the *rnhAB* mutant (Figure 2F), indicating epistasis of the *rnhAB* defect over the *recBC* defect.

We also measured DSB accumulation with pulsed-field gels in the *rnhAB*, *recBC*(Ts) and *rnhAB recBC*(Ts) strains exposed to 2, 16 or 36 J/m^2^ of UV doses, followed by 2 h of post-irradiation recovery in the growth medium at 37°C. We confirmed the same level of fragmentation after 16 and 36 J/m^2^ of UV in the *recBC* mutant (12), detected low level chromosome fragmentation in the *rnhAB* double mutant (as expected, because the strain is DSB-repair proficient (18)) and found no differences in the chromosome fragmentation between the *recBC* and *rnhAB recBC* mutants at any UV dose (Figure 2G). We conclude that the putative UVRLs in the *rnhAB* mutant are not resolved into double strand breaks, at least within the first 2 h after UV. The possibility that *formation* of UVRLs itself could depend on RecBC was tested later (Figure 4); initial UVRLs were found to form independently of RecBC.

### Post-UV DNA synthesis recovery is inhibited in the *rnhAB* mutants

Since UVRLs do not kill via chromosome fragmentation, we measured DNA synthesis rates in growing versus UV-irradiated cultures to test weather UVRLs inhibit replication restart after UV. The rate of DNA synthesis in the unirradiated *rnhAB* mutant cultures is about two times slower than in the WT cultures (Figure 2H, left), likely reflecting the 40% viability of the *rnhAB* mutants (18). After sublethal doses of UV irradiation, DNA synthesis in WT cells is known to be blocked for about one generation, until most PDs are removed by NER,—and then resumes at a slightly faster rate (6,7). We compared kinetics of the DNA synthesis restart after 36 J/m^2^ UV in three strains: WT, *uvrA* and *rnhAB* mutants. The WT culture behaved as expected: its DNA synthesis was blocked for 30 min, then suddenly resumed (Figure 2H). Again as expected, in the *uvrA* mutant, the post-UV DNA synthesis never recovered (Figure 2H), reflecting the inability of this mutant to remove PDs. In the *rnhAB* mutant, the post-UV DNA synthesis was blocked for 50 min (about twice as long as in WT cells), but then recovered from 50 to 90 min, only to level off after 120 min and to continue at this reduced level (Figure 2H). We concluded that in the *rnhAB* mutants, delayed and incomplete recovery of the post-UV DNA synthesis must reflect formation of UVRLs, which inhibit progress of replication forks, but without causing chromosome fragmentation.

### UVRLs are formed by transcription due to PDs in DNA

**Figure 3.**
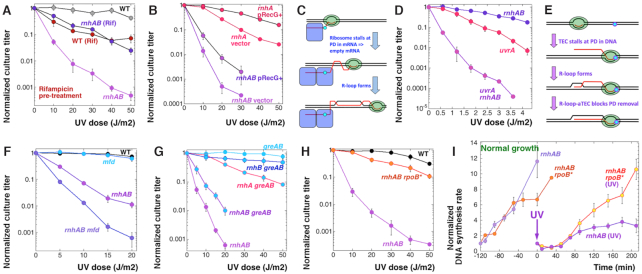
Genetic analysis suggests that UV-induced R-loops in the *rnhAB* mutants interfere with the post-UV DNA synthesis. (**A**) Rifampicin pretreatment (100 μg/ml for 5 min just before the UV-irradiation, at which time rifampicin is removed). The strains are: WT, AB1157; *rnhAB*, L-416. (**B**) The effect of increasing the copy number of RecG dsDNA pump. The strains are: *rnhA*, L-413; *rnhAB*, L-416. The plasmids are: vector, pBluescript; pRecG+, pSRK1*RecG*. (**C**) A scheme of R-loop formation via PD in mRNA. DNA duplex is shown as a pair of black lines; mRNA transcript as a red line. Green oval with a DNA bubble inside, transcribing RNA polymerase; big blue rectangles with rounded corners, ribosomes; small cyan circle, pyrimidine dimer. (**D**) Synergistic UV-sensitivity in the *uvrA rnhAB* mutant. The strains are: *rnhAB*, L-416; *uvrA*, SRK303; *uvrA rnhAB*, L-417. (**E**) A scheme of R-loop formation via PD in template DNA. (**F**) Effect of inactivation of transcription-coupled repair on UV-sensitivity of the *rnhAB* mutant. The strains are: WT, AB1157; *mfd*, L-505; *rnhAB*, L-416; *rnhAB mfd*, L-508. (**G**) Effect of the *greA greB* mutations on the UV-sensitivity of the *rnhAB* mutant. The strains are: *rnhAB*, L-416; *greAB*, L-474; *rnhA greAB*, L-479; *rnhB greAB*, L-477; *rnhAB greAB*, L-478. (**H**) The effect of the rpoB*35 mutation. The strains are: WT, AB1157; *rnhAB*, L-416; rnhAB rpoB*, L-416-33. (**I**) Recovery of DNA synthesis after 36 J/m^2^ of UV (administered at time = 0) in the rnhAB rpoB* mutant, compared to *rhnAB* (the latter repeated from Figure 2H). The DNA synthesis rates in unirradiated cultures are shown as controls. The strains are: *rnhAB* (L-416) and rhnAB rpoB* (L-416-33).

The inability of the *rnhAB* mutants to continue the restarted DNA synthesis after UV could reflect formation of R-loop-aTECs (18,30), which permanently stall replication forks in the head-on orientation in the absence of RNase H (31,32). We tested the contribution of transcription to the UV sensitivity of the *rnhAB* mutant by pretreating cells with rifampicin, which blocks transcription initiation, while allowing the ongoing transcription to finish (47). A 5 min pretreatment of growing cultures with 100 μg/ml rifampicin and its subsequent removal before UV-irradiation made the wild type cells mildly sensitive to UV, most likely via the residual rifampicin interference with SOS-induction (48) (Figure 3A). In contrast, rifampicin pretreatment elevated UV resistance of the *rnhAB* mutant to the (rifampicin-reduced) level of the wild type strain (Figure 3A), strongly implicating transcription after UV in the formation of lethal UVRLs. Such rifampicin pre-treatment does not change the UV-sensitivity of *recBC* or *recF* mutants (Supplementary Figure S4A), arguing for its specific effect in the *rnhAB* mutant, but it does alleviate the *ruvABC* mutant, again implicating transcription in its UV-sensitivity.

In the RNase H-deficient mutant, transcription likely interferes with replication via formation of R-loops. To see if R-loops could be a part of UVRLs, we overproduced the RecG, a duplex DNA pump with some R-loop disassembling activity (49), whose overexpression in *recBC*, *recF* or *ruvABC* mutants had either no or small effect (Supplementary Figure S4B). Overexpression of RecG from a plasmid indeed improved UV resistance of both the *rnhAB* double and the *rnhA* single mutants to a similar extent (Figure 3B), suggesting that R-loops indeed participate in UVRLs. But then how could PDs in DNA cause formation of R-loops, if the plasmid-nicking assay indicates normal kinetics of DNA-PD removal in the *rnhAB* mutants (Figure 1DE)?

Since ribosome-free mRNA stimulates R-loop formation (50), and since ribosomes stall at mRNA lesions (51), UV could induce R-loop-aTECs if ribosomes stall on *PDs in nascent mRNA*, yielding ribosome-free mRNA behind TECs (Figure 3C). This logic predicts that inhibition of translation elongation with chloramphenicol treatment before UV irradiation would further exacerbate UV-sensitivity of the *rnhAB* mutants. However, contrary to this expectation, we found no additive effect of chloramphenicol on the UV-sensitivity of the *rnhAB* mutants (Supplementary Figure S5A). Moreover, we observed an extreme UV-sensitivity of the *uvrA rnhAB* triple mutant: at UV doses as low as 0.6 J/m^2^, when *rnhAB, uvrA, uvrA rnhA* and *uvrA rnhB* strains are still fully UV-resistant, the triple mutant is already killed almost 20-fold (Figure 3D, Supplementary Figure S5B). This synergy of the *uvrA* and *rnhAB* defects strongly argues against ribosome stalling at PDs in mRNA, since UvrA protein recognizes PDs only in the context of duplex DNA (52). Synergy of two mutations inactivating distinct enzymes usually means that one of the enzymes ‘repairs/removes’ a particular damage, while the other one ‘prevents formation’ of the same damage (53). Since overexpression of UvrA failed to relieve UV sensitivity of the *rnhAB* mutant (Figure 1C), and therefore removal of UVRLs must be the function of RNase H enzymes, it follows that NER must prevent UVRL formation by excising PDs from duplex DNA.

### UVRLs must be R-loops formed at stalled Transcription Elongation Complexes

Therefore, it must be transcription-elongation complexes (TECs) blocked by PDs in template DNA that initiate formation of R-loop-aTECs (Figure 3E). RNA pol II stalling in eukaryotic cells was proposed to induce R-loops behind, after spliceosome eviction from mRNA (54). Our earlier observation that UvrA overexpression does not improve UV resistance of the *rnhAB* mutant (Figure 1C) suggests that PDs that cause UVRLs are inaccessible to NER,—for example being masked by stalled RNA polymerases (Figure 3E). Mfd protein promotes TCR by removing stalled RNA polymerase from the blocking PD and recruiting UvrAB to the now accessible PD (4). Such Mfd action predicts that the *mfd rnhAB* mutants should be more sensitive to UV. By themselves, *mfd* mutants are not UV sensitive (Figure 3F); however, the *mfd* defect indeed exacerbates the UV sensitivity of the *rnhAB* mutants (Figure 3F), supporting the idea that PD-stalled RNA polymerases contribute to UVRL formation.

One more process that removes stalled R-loop-aTECs is the long-range RNA polymerase backtracking by the UvrD helicase (55). GreA and GreB are elongation-processivity factors that minimize this UvrD-promoted RNA polymerase backtracking by restarting transcription (55). This logic predicts that the *greA* and *greB* defects, via eliminating control over backtracking, should make RNA polymerase vulnerable to UvrD removal and therefore should relieve the UV-sensitivity of *rnhAB* mutants. We found that *greA greB rnhAB* mutant is indeed more UV-resistant than the *rnhAB* mutant (Figure 3G), further supporting the idea that UVRLs are R-loop-aTECs.

If UVRLs are R-loop-aTECs, whose strength reflects the high stability of transcription complex, then destabilizing stalled RNA polymerase in *E. coli* with the rpoB*35 mutation (56) should suppress the UV sensitivity of the RNase H-deficient cells. Indeed, we found the rpoB*35 mutation to be the strongest suppressor of UV-sensitivity of the *rnhAB* mutant (Figure 3H). Moreover, even though the post-UV lag of the chromosomal DNA synthesis in the rhnAB rpoB*35 mutant is as long as in the *rnhAB* mutant, its replication completely recovers after 60 min, in contrast to *rnhAB* mutant (Figure 3I). At the same time, the rpoB*35 defect neither accelerates the repair of PD dimers by global NER (Figure 1E), nor reduces the density of DNA-rNs in the *rnhAB* mutants (Supplementary Figure S6).

Thus, our UV-sensitivity and DNA synthesis rate data strongly argue for the formation of UV-induced R-loop-aTECs at PDs in DNA, with RNase HI and RNase HII being the main enzymes to remove them (Supplementary Figure S7). Moreover, processes destabilizing transcribing RNA polymerases and R-loops alleviate the UV-sensitivity of *rnhAB* mutants, further supporting this scenario (Supplementary Figure S7). Importantly, the idea of PD-dependent R-loop-aTECs (Figure 3E) generates two testable predictions about chromosomal DNA in the UV-irradiated *rnhAB* mutants: (i) it should accumulate RDHs representing R-loop-aTECs; (ii) it should preserve a small fraction of NER-resistant PDs (presumably those that cause R-loop-aTEC accumulation).

### UV-induced transcriptional RDHs accumulate in *rnhAB* mutants

**Figure 4.**
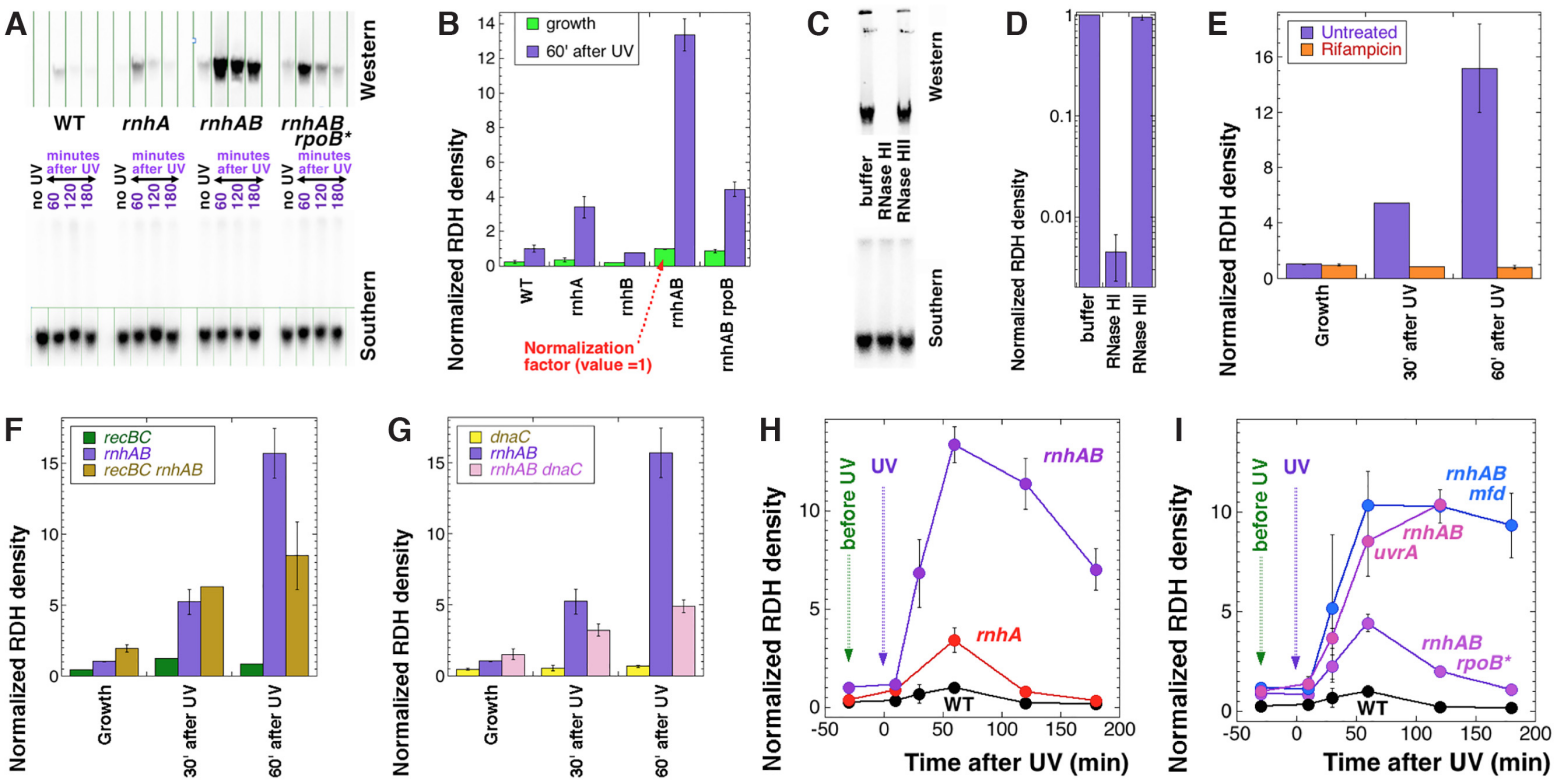
Accumulation of RNA:DNA hybrids in the chromosomal DNA after UV. Cell cultures were irradiated with 36 J/m^2^, then recovered and analyzed at the indicated times. (**A**) The chromosomal RDH assay: western blot with S9.6 antibody (top), the corresponding southern blot as a loading control for DNA amount (bottom). The strains are: WT, AB1157; *rnhA*, L-413; *rnhAB*, L-416; rnhAB rpoB*35, L-416-33. (**B**) The relative amount of RDHs in the chromosomal DNA during normal growth (green bars) versus 60 min post-UV (purple bars). The RDH values were derived by dividing the western S9.6 signal by the corresponding southern signal, then normalized to the RDH value of unirradiated *rnhAB* mutant (L-416), as quantified from gels like in ‘A’. (**C**, **D**) RDH structures from the *rnhAB* mutant (L-416) are sensitive to RNase HI, insensitive to RNase HII. C. A representative western-southern couple. D. Quantification of RDH signal stability against RNase HI versus RNase HII. Note the semi-log scale, to stress how little signal survives RNase HI treatment. (**E**) Rifampicin-sensitivity of the RDH signal in *rnhAB* mutant (L-416). (**F**) RecBCD is required for the maximal increase of the RDH signal. The strains are: *recBC*(Ts), SK129; *rnhAB*, L-416; *recBC*(Ts) *rnhAB*, L-476. (**G**) DNA synthesis is required for the maximal RDH signal. The strains are: *dnaC*, L-393; *rnhAB*, L-416; *rnhAB dnaC*, L-504. (**H**, **I**) Evolution of the RDH signal after 36 J/m^2^ of UV in the strains indicated. Exception: the UV dose for the *rnhAB uvrA* mutant was 4 J/m^2^. The strains are: WT, AB1157; *rnhA*, L-413; *rnhAB*, L-416; rnhAB rpoB*35, L-416-33; *rnhAB mfd*, L-508; *uvrA rnhAB*, L-417.

To identify the predicted UV-induced RDHs in the *rnhAB* mutants, we probed chromosomal DNA with the RDH-specific antibodies S9.6 (57). To minimize the potential RNA contamination in our assays, we removed the bulk of RNA with RNase A treatment during cell lysis and ran the DNA samples in two identical agarose gels, followed by electric transfer of the nondenatured samples from one gel to nylon membrane for Western analysis with S9.6 antibodies (Figure 4A, top). To quantify RDH density, we normalized the S9.6 signal in the chromosomal DNA band to the corresponding chromosomal DNA signal from Southern analysis of the other gel (Figure 4A, bottom). Since the S9.6 signal is barely detectable in the RnhA+ strains, yet is readily detected in the growing *rnhAB* mutants, for a reliable comparison between strains and experiments we normalized each RDH density value within the experiment to the RDH density value of the growing *rnhAB* culture (Figure 4B, E–I).

First, we compared the RDH density in growing cells to the one from UV-irradiated cells, recovered post-UV for 1 h (Figure 4B). RDH signal is detected even in unirradiated growing cultures, with the maximum value in the *rnhAB* mutants (Figure 4B, the green bars). After 1 h of post-UV recovery, RDH density increases in all strains: ∼4-fold in WT and *rnhB* single mutant cells, 9-fold in the *rnhA* single mutant, ∼13-fold in the *rnhAB* double mutant, ∼5-fold in the rnhAB rpoB* triple mutant (Figure 4B, green versus purple bars). We conclude that, in various *rnh* mutants, their UV-sensitivity correlates with both the relative and the absolute accumulation of RDHs.

The UV-induced RDH signal from genomic DNA is removed by in vitro treatment with RNase HI, but not with RNase HII (Figure 4CD), suggesting its R-loop nature. At the same time, UV-induction of RDH signal is completely blocked by rifampicin (Figure 4E), indicating its generation as a result of transcription. This RNase HI-sensitivity of the RDH signal (also see Supplementary Figure S8), its dependence on transcription (Figure 4E and Supplementary Figure S9A), as well as its distribution over the chromosome (Supplementary Figure S9C) and resistance to high temperatures (Supplementary Figure S10) are all consistent with its being extended RDHs, likely long R-loops (see the Supplement for a complete description of this characterization).

### Accumulation of RDHs depends on restart of DNA replication

Since UV-irradiated *recBC rnhAB* mutant cells failed to induce additional chromosome fragmentation over the *recBC* mutant background (Figure 2G), there was a possibility that the *recBC* defect interferes with formation of UVRLs in the *rnhAB* mutant, yielding lower RDH density. However, we found no difference between RDH signals between the *rnhAB* and *recBC rnhAB* mutants, either in growing cells or 30 min after UV, although the RDH signal 60 min past UV was indeed ∼2-fold lower in the *recBC* background (Figure 4F). Since the post-UV replication recovery happens around this time in the *rnhAB* mutant (Figures 2H or 3I), and since RecBCD enzyme is important for post-UV replication recovery (12), the decrease in RDH signal due to the *recBC* defect suggested involvement of DNA replication in generation of the maximal UV-induced RDH signal in the *rnhAB* mutants. Indeed, blocking replication (re)initiation with the *dnaC* mutation (58) decreases RDH signal at 60 min post-UV (Figure 4G),—confirming that active replication forks are required to generate maximal UV-induced RDH signal.

To find the time of maximal accumulation of RDH signal, we performed the time course in various mutants (Figure 4HI). We found that: (i) although there is no additional RDH signal at 10 min after UV, the significant increase in RDH signal at 30 min post-UV happens when no DNA synthesis is detected yet (*cf*. Figure 2H); (ii) maximum RDH signal in all strains is at 60 min post-UV—by this time DNA replication is either fully recovered (in WT cells, Figure 2H) or starts recovering (in the *rnhAB* and rnhAB rpoB35* mutants, Figure 3I),—supporting the idea that replication restart is required to amplify RDH; (iii) once at the maximal level, RDH signal remains relatively stable in the *rnhAB* and *rnhAB mdf* mutants for 1 or 2 h, while decreasing to the background levels in the WT, *rnhA* and rhnAB rpoB*35 strain (Figure 4HI). Importantly, rpoB*35 mutation decreases both formation (at 30 min post-UV) and accumulation (at 60 min post-UV) of RDHs and also facilitates their removal (at 120 min post-UV) (Figure 4I), which is in agreement with the UV viability and rifampicin treatment data, and shows that stable (WT) RNA polymerase is critical for the formation and stability of UV-induced RDHs.

Finally, the maximal RDH signal in the *rnhAB* mutants after 36 J/m^2^ of UV requires active NER, as it is reduced several times in the *rnhAB uvrA* mutant (Supplementary Figure S11). However, at 9× lower UV dose of 4 J/m^2^, the RDH signal increase in the *rnhAB uvrA* triple mutant becomes similarly dramatic (Figure 4I), suggesting that, by removing PDs, NER promotes RDH signal increase indirectly, likely by relieving UV-inhibition of DNA synthesis (12).

### A small fraction of PDs is resistant to NER in the *rnhAB* mutants

**Figure 5.**
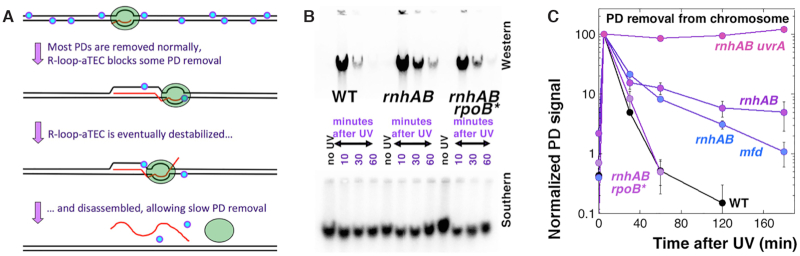
Removal of PDs from the chromosomal DNA of the *rnhAB* mutants. Cell cultures were irradiated with 36 J/m^2,^ then recovered and analyzed at the indicated times. (**A**) A scheme of how R-loop-aTECs could mask some PDs from NER. (**B**) The chromosomal DNA PD detection assay—western blot with KTM53 PD-specific antibody signal (top), and the corresponding southern blot as a loading control for the DNA amount (bottom). The strains are: WT, AB1157; *rnhAB*, L-416; rnhAB rpoB*, L-416-33; *uvrA rnhAB*, L-417; *rnhAB mfd*, L-508. (**C**) PD removal from the chromosomal DNA, quantified from gels like in ‘B’. PD values for each time point were derived by dividing western signal by the southern signal and then normalized to the PD value of the 5 min post-UV treatment. The 0 min point is taken before UV.

Our genetic and physical data so far strongly support the idea that, after UV exposure, the ongoing transcription spawns accumulation of RDHs, implying they are R-loop-aTECs stalled at pyrimidine dimers (Figure 3E). In the *rnhAB* mutants, these R-loop-aTECs cannot be removed and limit the post-UV recovery of DNA replication. This scenario predicts that, while the bulk of PDs in the *rnhAB* mutants is removed without problem, the few PDs which cause the RDH increase should be masked from NER, by either stalled RNA polymerases or by the associated R-loops (Figure 5A). To test the prediction that a small fraction of PDs would be removed slowly from the chromosomal DNA of UV-ed *rnhAB* mutant cells, we determined kinetics of PD repair in WT, *uvrA*, rnhAB, rnhAB rpoB35* and *rnhAB mfd* mutants. In contrast to our previous plasmid-based measurement of the global NER capacity (Figure 1DE), this time we measured PD removal in the chromosomal DNA by anti-pyrimidine dimer-specific antibodies (PD-antibodies) (Figure 5B, top).

The PD-antibody signals were normalized to the corresponding DNA signals (Figure 5B, bottom) to calculate PD-densities. The PD-density from the 5 min post-UV sample was taken for 100% to calculate subsequent PD disappearance due to repair at 30, 60, 120 and 180 min (Figure 5C). Controls show either complete removal of PDs within 60 min in WT cells or PD persistence in the NER-negative (*uvrA*) mutants (Supplementary Figure S12A). The *rnhAB uvrA* mutant shows no PD removal either (Figure 5C). At the same time, in both the WT and rnhAB rpoB35* cells more than 90% of the chromosomal PDs are removed by 30 min (kinetics of PD removal is the same in the rnhB rpoB* RNase HI+ mutant (Supplementary Figure S12B versus Figure 1E)), while only background signal is detected by 60 min post-UV. In contrast, in the *rnhAB* and *rnhAB mfd* mutants, PD repair slows down after 30 min post-UV, with ∼10% of all PDs still persisting by 1 h and ∼5% still detectable by 2 h (Figure 5C).

We conclude that: (i) in the *rnhAB* mutants, 5–10% of PDs in the chromosomal DNA are removed slowly, if at all; (ii) in this and also in the *rnhAB mfd* mutants, at 60 min post-UV recovery, the highest RDH signal correlates with retention of ∼10% PDs in the chromosomal DNA (Figures 4HI versus 5C); (iii) in the 30–60 min interval in these two mutants, the gradual PD removal correlates inversely with the RDH accumulation, suggesting that R-loops expand, while PDs are slowly yet continuously removed; (iv) the rnhAB rpoB35* mutant repairs PDs like WT,—apparently because of the unstable RNA polymerase. Interestingly, up to 30 min post-UV PD repair proceeds at similar rates in all NER+ strains (Figure 5C),—yet after that time the *rnhAB* mutants restart post-UV replication and at the same time slow down PD removal,—suggesting it is the arrival of replication forks that slows down removal of the remaining PDs; (v) no Mfd-dependent PD removal is observed in the *rnhAB* mutant.

### RDH fraction is enriched for PDs

**Figure 6.**
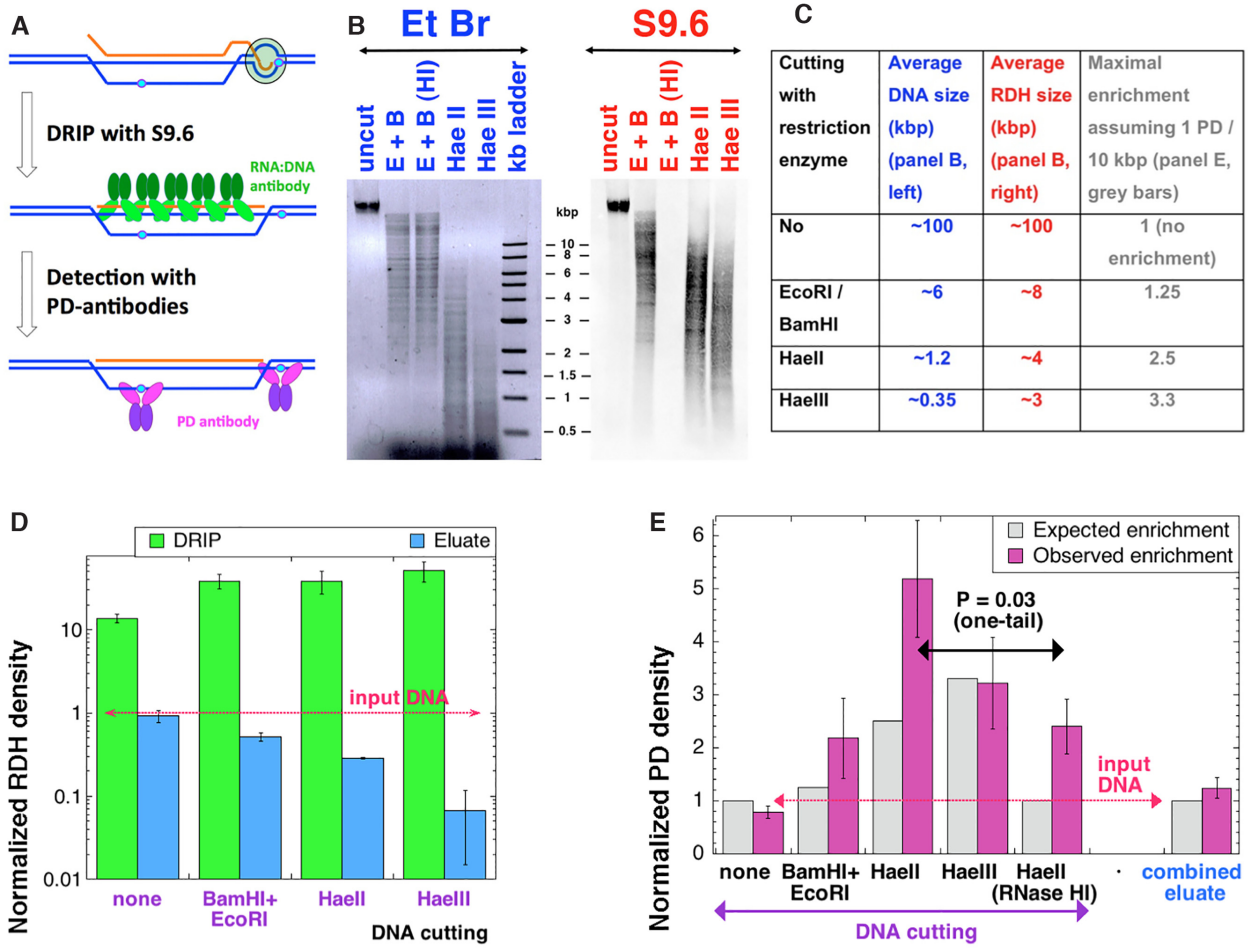
Co-enrichment of RDHs and PDs in the *rnhAB uvrA* mutant. (**A**) A scheme of co-enrichment. The isolated and enzymatically-digested DNA undergoes DRIP with S9.6 (the green antibodies), after which PDs are measured in the RDH-enriched DNA using PD-specific antibody. (**B**) Chromosomal DNA isolated from the *rnhAB uvrA* mutant (L-417) 1 h after irradiation with 8 J/m^2^ UV, cut with various restriction enzymes (E+B, EcoRI + BamHI; HI, RNase HI), run on a 0.8% agarose (the inverted EtBr-stained image of the gel is shown on the left), electric-transferred to the membrane and hybridized with S9.6 antibodies against RDHs (right). (**C**) Cutting versus the expected maximal enrichment, from the actual average length of RDHs (panel B) and the average PD density of 1 PD per 10 kb after 8 J/m^2^ of UV. (**D**) DRIP enriches for RDHs in DNA. The RDH density was determined in the DRIP fraction, the corresponding eluate fraction, and in the original INPUT fractions (the latter was used for normalizing the first two to calculate enrichment). Note the logarithmic scale of Y-axis. (**E**) DRIP enriches for PDs. The density of PDs was determined in the DRIP, the eluate and the input fractions from ‘D’ and then normalized to the input values (purple bars). Since all the eluates showed the same density of PDs (as expected), they were all averaged together. Theoretically-expected enrichment based on the density of PDs and on the average sizes of RDH distributions in panel C is shown by gray bars for comparison.

If some PDs stall transcribing RNA polymerases, thus avoiding detection by NER and also inducing RDH formation behind stalled TECs, — these NER-resistant PDs should be in proximity of the RDHs whose formation they have instigated (Figure 5A). If, so, enriching chromosomal DNA of the UV-irradiated *rnhAB* mutants for RDHs should also enrich this DNA for PDs (Figure 6A).

To test the idea of PD proximity to UV-induced R-loop-aTECs, we performed DNA:RNA immunoprecipitation (DRIP) of the chromosomal DNA isolated from *rnhAB* mutant 60 min post-UV, at the maximum of the RDH signal (Figure 4H). The recovered DRIP fraction was analyzed for both RDH and PD densities by dividing the antibody signals by the corresponding DNA signal, and further normalizing it to the RDH and PD densities of the input DNA, to calculate the enrichment factor. As a negative control, the same analysis was applied to the flow-through fraction of DNA that *failed* to bind to S9.6 antibodies in the DRIP protocol (we call it ‘eluate’), in which RDH signal *depletion* was expected.

We started DRIP enrichment with the *rnhAB* mutant irradiated with 36 J/m^2^, but detection of the remaining PD signal at 60 min post-UV was unreliable. Therefore, we switched to the *rnhAB uvrA* strain exposed to either 4 or 8 J/m^2^ UV (Figure 4I), to stabilize PDs. However, since in this excision-deficient strain, PDs were expected to be present in both the RDH-containing DRIP fractions and the eluate fractions, this made PD enrichment sensitive to the size of RDHs. Therefore, we performed DRIP analysis with the chromosomal DNA fragments of various sizes, as illustrated by the agarose gel (Figure 6B, left), with the logic explained in the table (Figure 6C).

Initially we assumed that R-loops were mostly short and within ∼1 kb of PDs that induced them. Therefore, we expected that cutting DNA to small ≤1 kb fragments would facilitate enrichment for PD signal after DRIP. The RDH size distribution was revealed by Western hybridization of the genomic DNA cut to various average fragment sizes (Figure 6B, C). Unexpectedly, we found that the RDH size distribution matched the actual fragment size distribution only for the EcoRI + BamHI cutting (Figure 6B, C). For HaeII and HaeIII cutting the average RDH signal size was much longer than the average DNA fragment size (Figure 6B, C). These longer-than-expected RDHs in the chromosomal DNA significantly limited the theoretically-possible enrichment factors for PDs in the DRIPped DNA (Figure 6C, the rightmost column).

As expected, the DRIP fraction was enriched for RDH, ∼10-fold for the uncut DNA and 30–50-fold for the digested DNA (Figure 6D). The enrichment of the DRIP fractions was validated by depletion of the RDH signal in the corresponding eluate fractions, so compared to the eluate, the overall enrichment (DRIP/eluate) was increased to 15–500, depending on the cutting (Figure 6D). Since only ∼1% of RDH signal survives RNase HI treatment (Figure 6B), we expected no enrichment after DRIP of the RNase HI-treated samples. However, S9.6 antibodies still provided some 15-fold enrichment of the RNase HI-treated DNA relative its eluate fraction (compared to 95-fold enrichment for untreated samples) (Supplementary Figure S13A), indicating an RDH-independent background of the procedure. Our attempts to reduce it by pre-treating the beads with glycogen or salmon-sperm DNA were unsuccessful, suggesting that it is coming from S9.6 antibody itself and is the intrinsic background of the DRIP procedure.

Next we measured enrichment for PDs in the DRIPped samples (Figure 6E). Since the eluate fractions of various digestions expectedly showed similar levels of ‘no enrichment’, we have combined them all in the ‘combined eluate’ value (Figure 6E, the far right). The observed PD enrichment by DRIP turned out to be somewhat higher than the theoretical expectations (Figure 6C), based on the idea of PD proximity to RDHs (Figure 6E, the purple bars versus gray bars). We found no PD signal enrichment without cutting, a 2-fold enrichment with EcoR + BamH cutting, a 3–5-fold enrichment with HaeII and HaeIII cutting, the latter two values being both significantly different from the (no) PD-enrichment value of the combined eluate (Figure 6E) (also see Supplementary Figure S13B, C for an alternative normalization yielding the same conclusion). RNase HI treatment of the chromosomal DNA before DRIP is expected to decrease the DRIP enrichment for both RDH and PDs. Indeed, after RNase HI treatment of the HaeII-cut DNA we found statistically-significant 2-fold decrease of PD-enrichment in the DRIPed fraction (Figure 6E). We conclude that enrichment for RDH with DRIP causes co-enrichment for PDs, indicating proximity of PDs to RDHs relative to the non-enriched genomic DNA.

### Co-enrichment of PDs with RDHs

Although DRIP did enrich for PDs in the chromosomal DNA of the *rnhAB* mutants, both the low theoretical limits of this enrichment (Figure 6C) and the technical issues of DRIP background made us seek a simpler and more robust procedure to further characterize and quantify the RDH-PD co-enrichment. We have noticed that, in the gel-separated HaeII-digested chromosomal DNA of UV-irradiated *rnhAB* mutants, the longest genomic DNA pieces (6–10 kb) both accumulate RDHs preferentially and repair PDs slower than the bulk of this DNA, which has a mode ∼1.2 kb (Supplementary Figure S14ABC). To test whether UV-induced RDHs indeed co-localize with the remaining PDs in the HaeII-digested genomic DNA, we partitioned these lane profiles, from wells to gel bottom, into 16 fractions, to calculate the density of the RDH or PD signals (Western / Southern) in individual fractions along the lane (Supplementary Figure S14D). The resulting density profiles for the *rnhAB* mutant 30 min post-UV all featured maxima in fraction #6 (8–10 kb) (Supplementary Figure S14D, the top graphs), signifying enrichment of this fraction for both RDHs and PDs relative to other fractions of the lane. We then calculated the RDH-PD co-enrichment profiles for individual experiments by multiplying the normalized RDH densities by PD densities of the correspondent fractions (Supplementary Figure S14D, the bottom graph).

**Figure 7.**
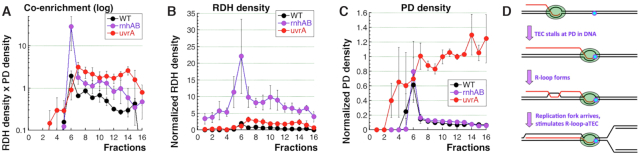
Co-enrichment profiles versus profiles of the RDH or PD densities. Strains: WT, AB1157; *rnhAB*, L-416; *uvrA*, SRK303. Growing cultures were exposed to 36 J/m^2^ of UV, and DNA was isolated before UV (log), as well as 5 min post-UV and 30 min post-UV. The density or enrichment profiles are for 30 min post-UV DNA samples. A scheme and description of quantification is in Supplementary Figures S14 and S15 and in Methods. (**A**) Co-enrichment profile of the *rnhAB* mutant compared with ones for the WT cells and the *uvrA* mutant. (Note the logarithmic Y-axis in this plot). The same plot, but with linear Y-axis, is in Supplementary Figure S14E. (**B**) Comparison of the RDH density profiles. (**C**) Comparisons of the PD density profiles. (**D**) The final model, according to which R-loop-aTEC formation from the initial R-loop requires arrival of a head-on replication fork.

Since we could not apply this calculation for the control WT strain at 60 min post-UV, because, even though it did have detectable RDHs (Figure 4ABH), all PDs were already removed (Figures 1E and 5C),—we limited this analysis to the time 30 min post-UV. Such quantification for WT cells shows an almost uniform co-enrichment across the lane with an average non-peak value of ∼ 0.5 (which can be taken for the procedure background in excision repair-proficient cells) and a small peak of ∼2.0 in fraction #6 (Figure 7A). In contrast, the *rnhAB* mutant shows a sharp co-enrichment peak of ∼29 in the same fraction #6 and the average non-peak co-enrichment of ∼1.0 (Figure 7A; also see the linear version of this plot in Supplementary Figure S14E). Thus, while the non-peak *rnhAB* profile shows 2× higher RDH-PD co-enrichment over the WT background, its peak fraction #6 features ∼15× higher co-enrichment. Interestingly, this peak co-enrichment of the *rnhAB* mutant was mostly driven by the RDH differences with the WT strain (Figure 7B), as their PD density profiles were similar (Figure 7C).

While the wild type cells provided the ‘low background’ of RDH-PD co-enrichment, the nucleotide-excision-deficient *uvrA* mutants, with their extremely high and evenly-distributed PD signal (Figure 7C) yet low and uniform RDH signal (Figure 7B and Supplementary Figure S11), should provide the ‘high background’ of RDH-PD co-enrichment. Indeed, the *uvrA* mutant developed no co-enrichment peak, but showed a co-enrichment plateau around the average of 2.0 (Figure 7A), which also coincided with the co-enrichment peak of fraction #6 in the WT cells. This confirms not only the ‘high background’ estimate for RDH-PD co-enrichment, but also the reality of ∼15× higher RDH-PD co-enrichment peak in the *rnhAB* mutant (Supplementary Figure S14E).

Calculations of co-enrichment, based on an alternative way to normalize fraction densities that reduces run-to-run variations, have reached the same conclusions (Supplementary Figure S15). Curiously, profiles in Figure 7C indicate that PD densities in fraction #6 are similar in all three strains, suggesting that PDs in this fraction are protected from excision in the excision-proficient cells. Since 60 min post-UV, PDs completely disappear in WT cells, while modestly decreasing in the *rnhAB* mutant (Figure 5C), their subsequent persistence in this mutant must be due to its much higher RDH density (Figure 7B). Overall, the sharp co-accumulation of RDHs with the slow-to-repair PDs in the longest size fraction in the HaeII-cut chromosomal DNA of UV-irradiated *rnhAB* mutants implies their physical co-localization and suggests that PDs are the cause of RDH accumulation.

## DISCUSSION

Ultraviolet irradiation (UV) is, perhaps, the best understood DNA-damaging treatment, and not only because it is the most frequent natural form of DNA damage, but mostly because it generates a narrow class of DNA lesions, primarily pyrimidine dimers (PDs), that are removed by the best-studied and ubiquitous form of repair, the nucleotide excision repair (NER) (52). UV is known to kill bacteria when there are too many UV-lesions in DNA (saturating excision repair) (8), or via irreparable chromosome fragmentation (saturating recombinational repair) (12). Our investigation of the unexpected UV-sensitivity of the RNase H-deficient mutant of *E. coli* revealed a novel lethal UV-induced DNA lesion of a complex nature that forms in three distinct stages (Figure 7D). The first stage is UV-induced PD formation in a coding strand of an expressed gene. At the second stage, this PD blocks the progress of a TEC, with two consequences: (i) excision repair of this PD is prevented; (ii) the nascent mRNA forms a small R-loop behind the stalled TEC (this step is postulated, as it is hard to detect). These presumably short original R-loops near PDs require something else to become the chromosomal lesions that would be able to inhibit normal DNA synthesis after UV. The third stage of the UVRL formation reveals itself by amplification of RDH signal, coincident with the post-UV replication restart—therefore we propose that it is the arrival of head-on replication forks, that turns the original short R-loops into long R-loop-anchored transcription-elongation complexes (R-loop-aTECs) (Figure 7D). Such R-loop elongation is expected from reports that head-on replication-transcription conflict promotes R-loop formation (31,32). It is speculated that the positive supercoiling between the two polymerases could induce compensatory gyrase action on the back of R-loop, generating hyper-negative supercoiling promoting R-loop elongation (30). We propose that such PD-induced long RDHs become lethal DNA structures in the RNase H-deficient mutants, making the affected DNA duplex impassable for the replication machinery.

### The long search for the right model

Before arriving at the current model (Figure 7D) explaining the surprising UV-sensitivity of the RNase H-deficient *E. coli* mutant that still has functional NER and recombinational repair, we experimentally tested and disqualified other models of the postulated UV-RDH relationship yielding lethal DNA lesions, UVRLs (Figure 1A). The first one was a simple possibility that UV-independent preexisting RDHs deplete either NER or recombinational repair, making the *rnhAB* mutant cells phenocopies of the, correspondingly, *uvr* or *rec* mutants. Our earlier observation that the *uvrA rnhAB* strain grows slower than *rnhAB* strain (18) did imply that NER helps repair RDHs formed during regular growth, and therefore NER could be distracted by them and become less efficient to repair PDs. However, normal kinetics of PD removal from bulk DNA (Figure 1DE) of the *rnhAB* mutants and their WT-sensitivity to various DNA damaging treatments (Supplementary Figure S1CDE) was inconsistent with them becoming NER- or recombinational repair-defective phenocopies.

The second model envisioned DNA polymerase translesion synthesis through PDs introducing R-patches across them, inhibiting subsequent DNA synthesis in both strands (Figure 2A). However, inactivation of both translesion DNA polymerases failed to alleviate UV-sensitivity of the *rnhAB* mutants, while the density of DNA-rNs after UV in these mutants failed to increase (Figure 2BC),—both observations being inconsistent with this model.

The third model, an aggravation of a milder scenario in Figure 2E, envisioned that aborted repair of a PD in the DNA strand of an R-tract results in replication fork explosion (Supplementary Figure S16), which forms irreparable double-strand breaks (40). However, its supposedly more frequent ‘sister’ scenario, replication fork collapse at the R-loop across the NER-generated ss-gap (Figure 2E), predicted not only synergy between UV-sensitivity of the *rnhAB* and *recBC* defects, but also more chromosome fragmentation in the *rnhAB recBC* mutants after UV. Neither effect was observed experimentally (Figure 2FG), disqualifying the model of irreparable double-strand breaks of Supplementary Figure S16.

The forth model was stimulated by our observation that recovery of the post-UV DNA synthesis in the *rnhAB* mutants is only partial (Figure 2H), suggesting a persistent interference, which we linked to UV-concurrent transcription generating R-loops (Figure 3AB). At first we envisioned stalling of translating ribosomes at PDs in mRNA, leaving the downstream mRNA empty and thus stimulating formation of R-loops, which should be stable in the *rnhAB* mutants (Figure 3C). However, PDs in mRNA predicted no additional UV-effect of the *rnhAB* inactivation in the highly UV-sensitive NER-deficient *uvrA* mutant, because NER removes PDs only from duplex DNA (52). Therefore, this attractive idea was disqualified by our finding of the synergistic interactions between the *uvrA* and *rnhAB* defects at the very low UV doses (Figure 3D), which instead strongly suggested that the culprits were PDs in DNA.

It is only when we envisioned TECs stalled at PDs in template DNA forming R-loop-aTECs (Figure 3E), that block subsequent replication through the region, had we finally arrived at a model whose predictions consistently matched our subsequent experimental observations. In particular, the model predicted that making RNA polymerase unstable should eliminate the UV-sensitivity of the *rnhAB* mutants (Figure 3H) and alleviate the inhibition of post-UV DNA synthesis (Figure 3I). It also predicted copious formation of UV-induced RDHs (Figure 4), a small fraction of repair-resistant PDs (Figure 5) and proximity of PDs to RDHs (Figures 6 and 7). But there was one important aspect of the mechanism of the lethal UV-induced R-lesions, revealed by our experimentation, that our model failed to anticipate,—which is discussed next.

### The role of DNA replication in RDH formation

Our initial model (Figure 3E), according to which R-loops formed due to TECs stalling at PDs, predicted new RDH formation right after UV. Indeed, it should take transcribing RNA polymerase in *E. coli* (moving at ∼50 nt/s (59,60)) about 20 seconds to traverse an average gene in *E. coli* (∼1 kb (61)). Even the longest genes or operons in *E. coli* should be transcribed in less than 5 min. If five more minutes is generously given for completion of R-loop formation,—then a significant RDH signal should be induced within 10 min after UV. Therefore, we were surprised to see absolutely no increase in RDHs 10 min after UV (Figure 4HI). The significant increase in RDHs was detected in another 20 min, 30 min post-UV, and reached its maximum at 60 min post-UV (Figure 4HI), as if post-UV RDH amplification depended on yet another factor, which was missing 10 min after UV and was still weak 30 min after UV.

The only factor missing during that time but restored 60 min after UV in the *rnhAB* mutants was DNA synthesis (Figure 2H). Indeed, inhibiting DNA synthesis restart with the *dnaC*(Ts) defect significantly reduces RDH amplification (Figure 4G), confirming replication forks as the third critical component in the formation of lethal UVRLs. It was reported for both bacteria and human cells that the head-on transcription-replication conflict stimulates formation of pervasive R-loops in the transcribed gene involved in the conflict (31,32). We propose that the same mechanism explains the UV-induced RDH accumulation, especially since excess of replication forks is expected in our cells. Indeed, besides the restarted regular forks, in the UV-irradiated *rnhAB* mutants there should be additional forks due to two types of stable DNA replication (62): (i) the constitutive one observed in the *rnhA* mutants; (ii) the inducible one observed in recombinational repair-proficient cells after any type of DNA damage and induction of SOS.

### Do R-loops cause double-strand breaks?

Whenever replication is inhibited, double-strand breaks tend to form (63,64), and we were initially surprised to find that inhibition of DNA replication after UV in the *rnhAB* mutants did not yield (additional) fragmentation (Figure 2G). In particular, this finding contradicts the widely-accepted view that replication fork collision even with protein-free R-loops generates DSBs (28,29). The key distinction could be accumulation of positive supercoiling.

It was argued before that transcription elongation complexes (TECs) with R-loops behind form stable structures (R-loop-aTECs) that in the *rnhAB* mutants could block head-on replication forks through accumulation of positive supercoiling, but without necessary generating double-strand breaks (30). Indeed, *E. coli gyrB* mutants with enhanced positive supercoiling did not require recombinational proteins for viability, suggesting that forks stalling by positive supercoiling does not result in double-strand breaks (65).

However, lethality and replication fork breakage *was* observed at the sites of transcription-replication collision in *recB* mutants in which *rrn* operons were inverted relative to *oriC*. Interestingly, under these conditions overexpression of RNase HI enzyme did not rescue lethality of the strain, suggesting that the conflict in this case did not involve R-loops, but was a direct collision between replication fork and RNA polymerases transcribing rDNA (66).

### Features of the UV-induced RDHs

To detect the UV-induced RDHs quantitatively, we used S9.6 RDH-specific monoclonal antibodies (57,67), normalizing the S9.6 signal of the chromosomal DNA band from western blot to the chromosomal DNA signal from Southern blot; since the latter varies depending on the strength of the probe, we further normalized the resulting RDH densities within every experiment to the RDH density in growing *rnhAB* cells (Figure 4). Although quantitative comparison of mostly qualitative Western signals is non-trivial (68), our RDH signal quantification proved accurate and reproducible, being able to reliably detect the differences between WT and *rnhAB* mutant strains even during normal growth, when the RDH density is low. An important contributor to this success could be our method of genomic DNA preparation, as not all DNA isolation methods are R-loop-friendly (57). Running genomic DNA in agarose gels instead of doing direct dot-hybridization also helped to reduce irreproducibility, likely by removing signal-generating or -inhibiting contaminations.

What is the nature of S9.6 signal that forms so abundantly in UV-irradiated RNase H-deficient cells? First, S9.6 antibodies besides RNA:DNA hybrids also detect dsRNA, although 5-times less efficiently (57,69). Our S9.6 signal is completely removed by in vitro treatment with RNase HI, but is mostly resistant to ssRNA and dsRNA-removing high-salt or low-salt treatments with RNase A (Supplementary Figure S8),—therefore it should represent RNA:DNA hybrids, either R-loops or R-tracts (Figure 1A, top). Indirectly, since we failed to detect UV-induced accumulation of R-tracts in plasmid DNA (Supplementary Figure S3), the chromosomal S9.6 signal most likely represents R-loops, rather than R-tracts. This tentative conclusion will have to be tested in the future.

The decreased strength and the complete disappearance with time of the UV-induced RDH signal in the *rnhA* single mutant contrast with high RDH signal accumulation and stability in the *rnhAB* double mutants, highlighting the role of RNase HII in RDH removal. Interestingly, as we find no RNase HII sensitivity of the RDH signal in the chromosomal band *in vitro*, in vivo removal of RDHs by RNase HII apparently requires help of other enzymes, for example DNA pol I, that should be able to utilize nick generated by RNase HII. Of note, since RNase HII in vitro does not attack RNA:DNA hybrids lacking RNA-DNA junctions (unless Mn^2+^ is substituted for Mg^2+^), the in vivo requirement for RNase HII inactivation for maximal accumulation of RDH signal suggests a non-R-loop nature of RDHs. However, it could simply be that the RNA strand of R-loop may have patches of incorporated DNA nucleotides long enough to be recognized by RNase HII as proper substrates; RNA polymerases are known to have a limited discrimination against DNA precursors (70,71).

The chromosomal RDH structures are thermally stable, since we observe complete RDH signal disappearance only at 90°C, when chromosomal dsDNA also completely denatures (Supplementary Figure S10C). Cutting chromosomal DNA with restriction nucleases reveals relatively smooth genomic distribution of the UV-induced RDH signal and estimated the average size of RDHs as 3–4 kb (Figure 6B), — much longer than we anticipated, but consistent with their thermal stability. The length of an R-loop could be estimated with whole-genomic sequencing after sodium bisulfate treatment to turn cytosines into uracils in the single-stranded DNA displaced by the R-loop. Clusters of genes in the range of 2–10 kb with the increased bisulfate sensitivity were identified even for the WT *E. coli* (50).

### The critical link between unexcised PD and RDH

The corollary of our model (Figure 5A) is the proximity of UV-induced RDHs to the PDs that caused them. To identify the R-loop-aTECs blocked at PDs we first followed kinetics of PD removal from the chromosomal DNA using PD-specific antibodies. While the *rnhAB* mutants removes PDs from plasmid with WT kinetics (by 60 min post-UV) (Figure 1DE), and PD removal from the chromosomal DNA in the rpoB35* rnhAB mutant and in WT cells is likewise complete by 60 min post-UV (Figure 5C), we found that 10% of PDs still remains by 60 min post-UV in the chromosomal DNA of the *rnhAB* and *mfd rnhAB* mutants (Figure 5C), when the RDH signal actively accumulates in these cells (Figure 4H, I), potentially linking the RDH signal accumulation with the lingering PDs.

We developed a detection procedure to directly test this logic (Figure 6A): using DRIP, we recovered the RDH-enriched DNA fragments, to subsequently analyze them for the density of PDs relative to either the input DNA, or the eluate DNA that failed to bind S9.6. The RDH-enriched fraction indeed showed the expected or higher PD densities relative to the eluate fraction, and both RDH and PD enrichments were reduced by RNaseHI treatment (Supplementary Figure S13A and 6E), supporting proximity of RDHs and PDs in the chromosomal DNA. In other words, even though UV-induced RDHs turned out to be quite long (lowering theoretically-possible enrichment), we still found DNA around them to be enriched for PDs, validating the proposed scheme (Figure 6A).

One weak point of the DRIP enrichment procedure is that it critically depends on not only the high specificity of S9.6 antibody for RDHs, but also on the absence of recognition of DNA with PDs; unfortunately, we found that both are not exactly true for this antibody. First, our DRIP still captures substantial amount of DNA even from WT cells, which have almost no detectable RDH signal (Figure 4A,B, H). Moreover, we could not reduce this background of non-specific dsDNA by pretreatment of protein-A beads with either glycogen or salmon-sperm DNA, suggesting that it is coming directly from S9.6 binding to DNA. Second, and even more troublesome for our objective, we found that such DRIP enrichment of control DNA from RNase H^+^ (WT) cells is further increased by UV-irradiation. Therefore, the observed small but significant RNase HI-resistant ‘enrichment’ of PD (Figure 6E) was unfortunate, but not exactly unexpected. There are multiple reports that S9.6 can precipitate non-R-loop DNA structures, reducing the specificity of DRIP (57,72).

We eventually solved the problem of co-enrichment detection differently, by size-fractionating RDH and PD signals of the HaeII-cut chromosomal DNA and by showing that accumulation of the RDH signal happens exactly in the same size fraction (6–10 kb) in which PDs are protected from excision (Figure 7). The procedure also reveals detectable RDH-PD co-enrichment in WT cells that happens in the same size fraction (Figure 7A),—but RDHs are actively removed in these RNase H-proficient cells, ultimately making the transcription-stalling PDs vulnerable to excision repair, which resolves the potentially-lethal situation.

### Conclusion

In this work, we present multiple lines of evidence to strongly implicate R-loop-aTECs as lethal R-lesions induced by UV in the *rnhAB* mutants, in two identifiable stages after the formation of PD. Indeed, the connection between R-loop and the PD has to be via transcribing RNA polymerase, while RDH amplification is induced only after arrival of replication forks (Figure 7D). Thus, instead of a novel yet simple structure (like R-tract), R-lesions after UV turn out to have a complex structure and a fittingly-complex mechanism of formation. From a different perspective, UV light, as a DNA-damaging agent, continues to surprise, first with pyrimidine dimers and their nucleotide-excision repair, then with chromosome fragmentation and its recombinational repair, now with R-loop-aTECs and their disassembly by RNase H enzymes. The next question in this line of research should be the chromosomal position of UV-induced RDHs,—addressed by genomic techniques.

## Supplementary Material

gkab147_Supplemental_File
