# Supplementary material for: Ultraviolet-induced RNA:DNA hybrids interfere with chromosomal DNA synthesis
Source: Nucleic Acids Res. 2021 Mar 10;49(7):3888–906. doi: 10.1093/nar/gkab147 (PMC8053090; doi:10.1093/nar/gkab147)
Supplement: gkab147_Supplemental_File [file gkab147_supplemental_file.pdf]

Supplemental Figures

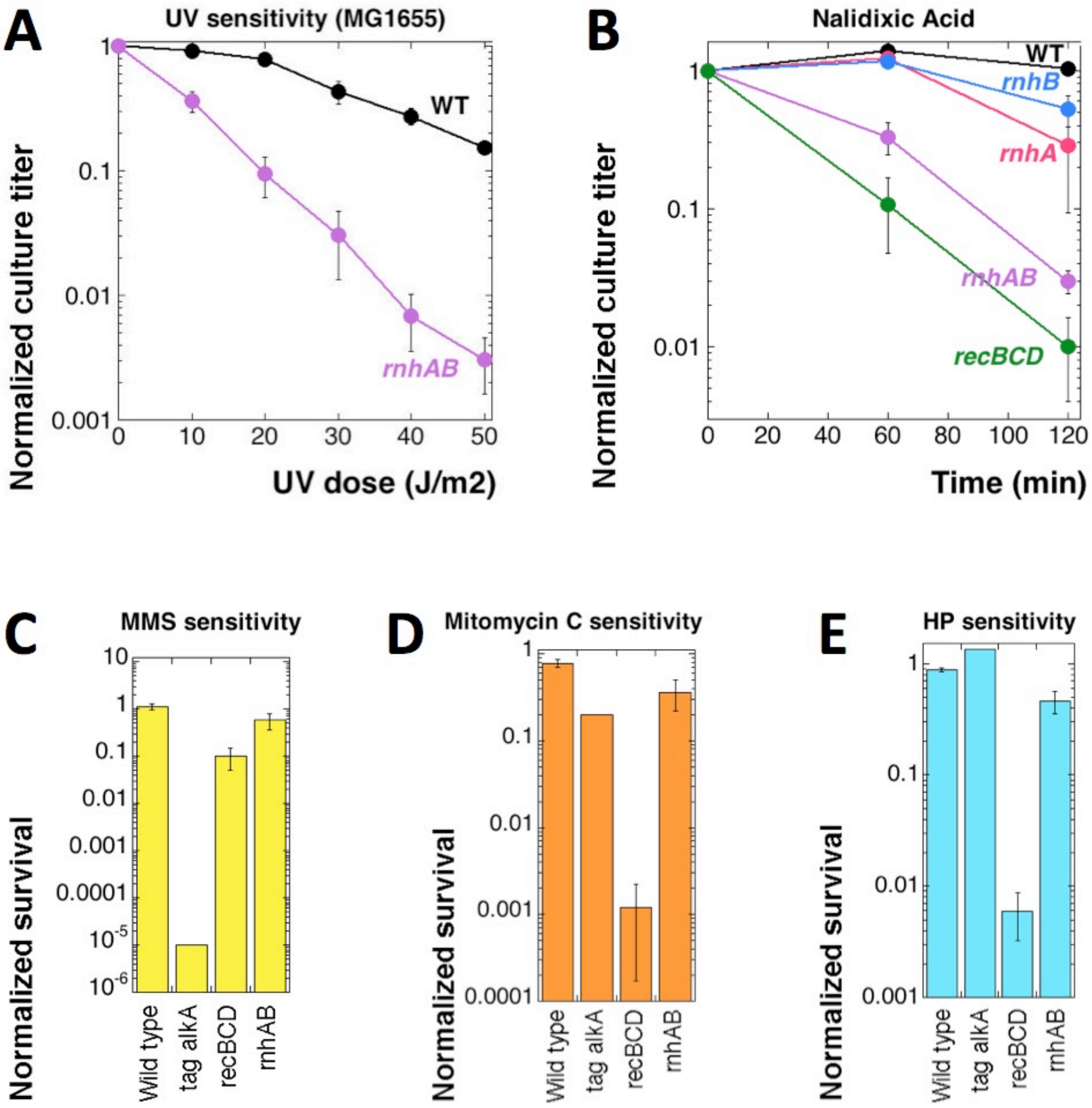

Fig. S1. Other backgrounds and DNA-damaging treatments. Except for Panel A, the strains are: WT, AB1157; Δ*rnhA*, L-413; Δ*rnhB*, L-415; Δ*rnhAB*, L-416; *tagA alkA*, GC4803; *recBCD*, JB1.

A. UV-sensitivity of the *rnhAB* mutant in the MG1655 background. The strains are: WT, MG1655; *rnhAB*, L-419.

B. Sensitivity to 30 μg/ml nalidixic acid at 37°C.

C. Sensitivity to 10 mM methanesulfonate (MMS) for 30 minutes at 28°C.

D. Sensitivity to 10 μg/ml mitomycin C for 30 minutes at 28°C.

E. Sensitivity to 10 mM hydrogen peroxide (HP) for 15 minutes at 28°C.

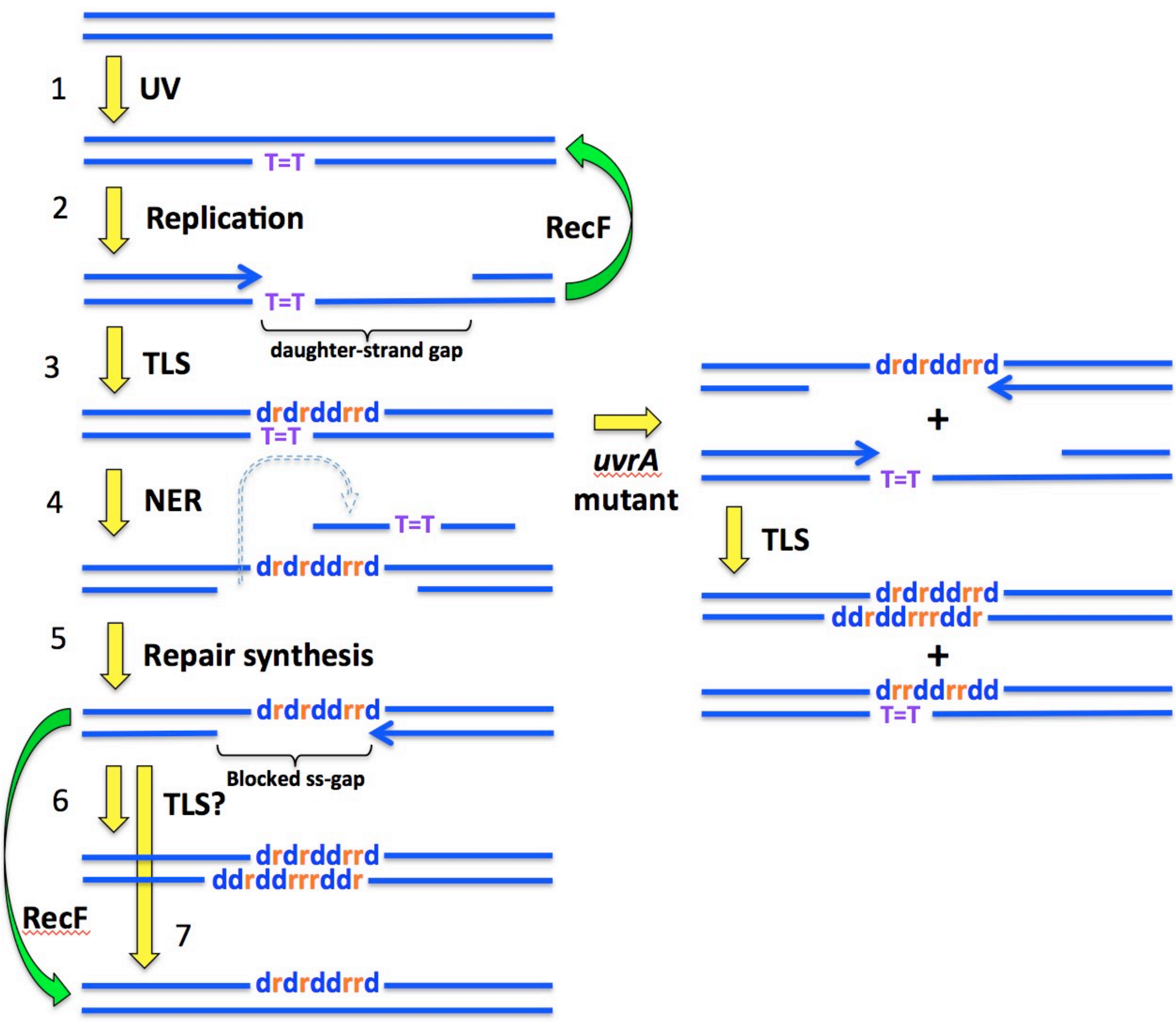

Fig. S2. The complete scheme explaining how translesion DNA synthesis could generate various two-strand R-lesions at PD. Blue lines, DNA strands; blue letters "d", DNA nucleotides; orange letters "r", RNA nucleotides; T=T, pyrimidine dimer.

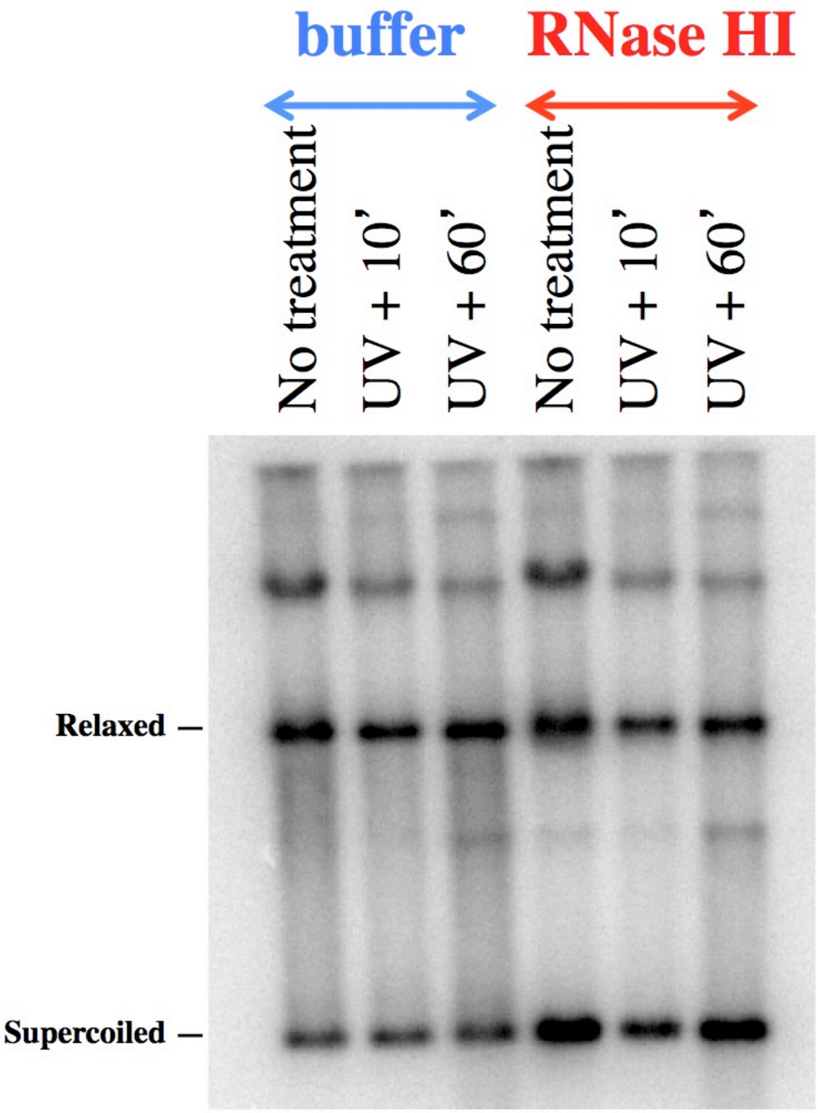

Fig. S3. No RNase HI dependent R-tracts detected in the plasmid (pEAK86) isolated from UV-irradiated *rnhAB* mutant (L-416) cells. The UV dose was 100 J/m^2, with subsequent shaking in the growth medium at 37°C for either 10 or 60 minutes.

Instead of causing plasmid relaxation, which would be indicative of R-tracts, RNase HI treatment reveals R-loops in these plasmid DNA preparations, especially in the "no treatment" sample, visible as the massive smear between the supercoiled and relaxed bands. After RNase HI treatment, the smear mostly disappears, as its plasmid material apparently goes into the supercoiled band, which increases several times. This is the expected behavior of R-loops: 1) they relax supercoiled DNA (so that R-loop removal tightens it back); 2) they are sensitive to RNase HI treatment.

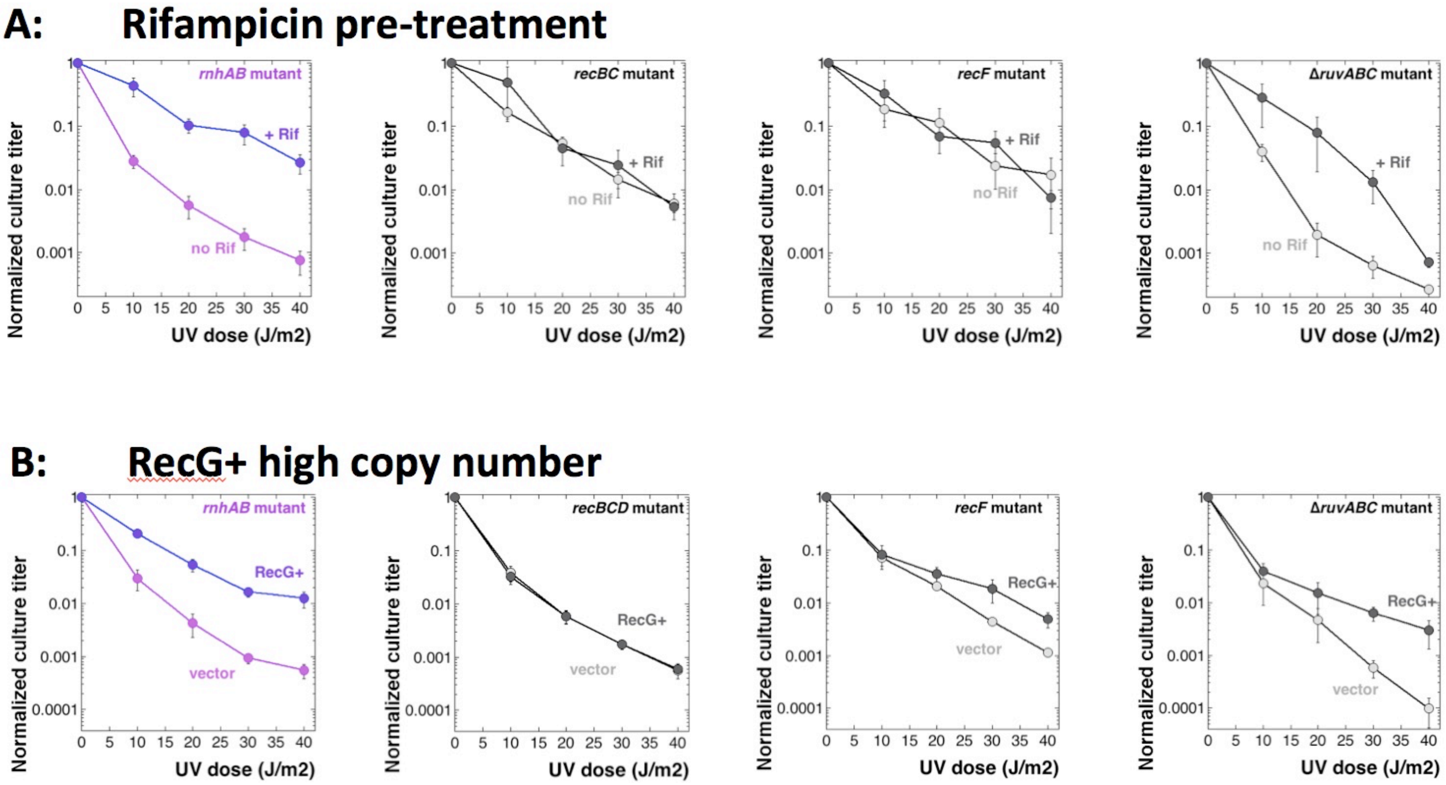

Fig. S4. Controls for rifampicin pretreatment and RecG+ increased copy number. The strains and plasmids are: *rnhAB*, L-416; *recBC*, SK129; *recBCD*, L-389; *recF*, AM3; Δ*ruvABC*, JJC754; vector, pBluescript; RecG+, pSRK*recG*.

A. Rifampicin pre-treatment. The left-most panel is an independent repetition of Fig. 3A.

B. Increased copy number of RecG+. The left-most panel is an independent repetition of Fig. 3B.

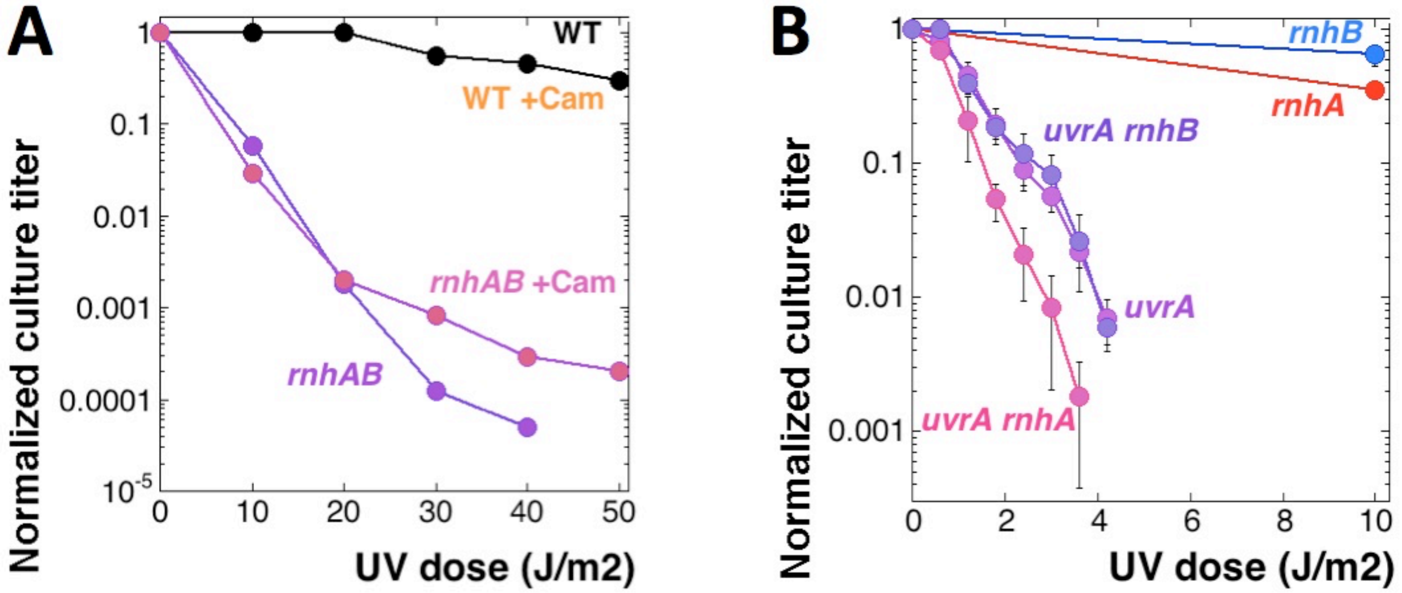

Fig. S5. UV-sensitivity: chloramphenicol pretreatment and the *uvrA* defect in combination with single *rnhA* or *rnhB* defects.

A. The effect of chloramphenicol pretreatment (200 μg/ml for 5 min) on UV-sensitivity of WT (AB1157) and *rnhAB* mutant (L-416).

B. Synergy in UV-sensitivity between the *uvrA* defect in NER and the *rnhA* defect, but not *rnhB* defect. The strains are: *rnhA*, L-413; *rnhB*, L-415; *uvrA*, SRK303; *uvrA rnhA*, L-414; *uvrA rnhB*, L-405

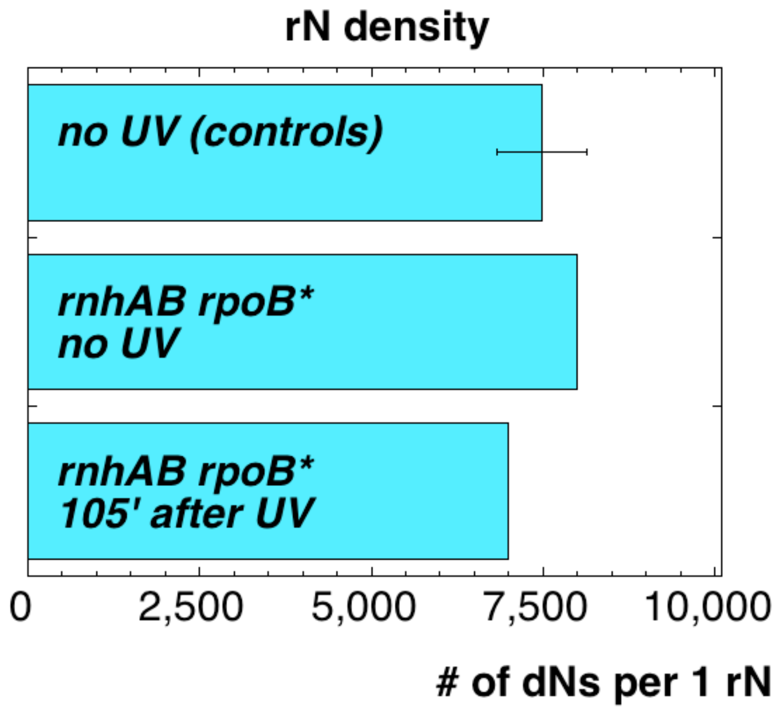

Fig. S6. The effect of the *rpoB** mutation on the DNA-rN density in the *rnhAB* mutant. The No-UV control value is from Fig. 2C. The *rnhAB rpoB** mutant strain is L-416-33 and the plasmid is pEAK86. UV dose was 40 J/m^2.

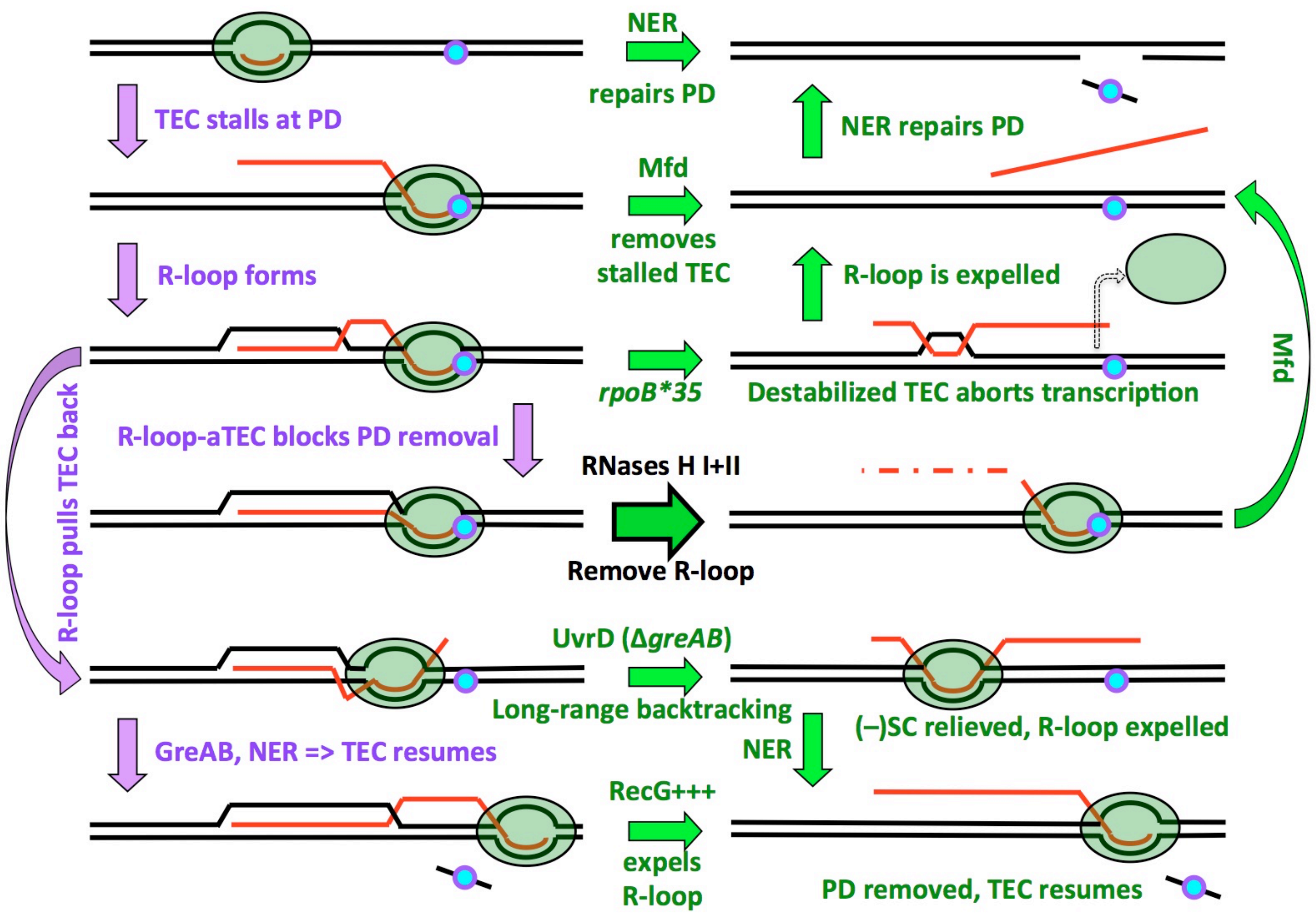

Fig. S7. A combined model of PD-stimulated R-loop formation: protagonists versus antagonists. Designations are like in Fig. 3CE. Purple arrows and text: pathways leading to lethal or potentially lethal UV-induced R-lesions. Green arrows and text: pathways of repair. TEC, transcription-elongation complex; R-loop-aTEC, R-loop-anchored TEC; NER, nucleotide-excision repair; PD, pyrimidine dimer; (–)SC, negative supercoiling.

Characterization of the UV-induced S9.6 signal in the chromosome

Besides RNA:DNA hybrids, S9.6 antibody also recognizes (5 times weaker) dsRNA, as was shown during its initial characterization (*1*) and confirmed in the recent DRIP-seq protocols (*2*). It should be pointed out that we detect the S9.6 signal only in the chromosomal DNA band, since we removed mRNA and rRNA with RNase A treatment during cell lysis, while tRNA is not recognized by S9.6 antibodies, as is illustrated in Fig. 6B.

We tested whether the observed UV-induced S9.6 signal represents transcription-generated RNA:DNA hybrids all over the chromosome in several ways. First, we verified that S9.6 signal is sensitive to the RNase HI treatment *in vitro*, but is resistant to RNase HI in the presence of 20 mM EDTA (Fig. S8A, lanes 3 and 4), confirming RNase HI specificity and purity. Further, the S9.6 signal is mostly sensitive to RNase A in a low salt buffer (in these conditions, RNase A attacks any form of RNA, including RNA:DNA hybrids), but is mostly resistant to RNase A in high salt buffer (when the enzyme attacks mostly ssRNA) (Fig. S8A, lanes 5 and 6). We conclude that the UV-induced S9.6 signal is RNase HI-sensitive and therefore the S9.6-detected structures have an RNA:DNA hybrid component. Interestingly, the S9.6 signal in the chromosome is resistant to the RNase HII treatment *in vitro*, whereas DNA-rNs in plasmid from the same *rnhAB* mutant are cleaved by RNase HII, causing plasmid relaxation (Fig. S8C, lanes 1 and 5).

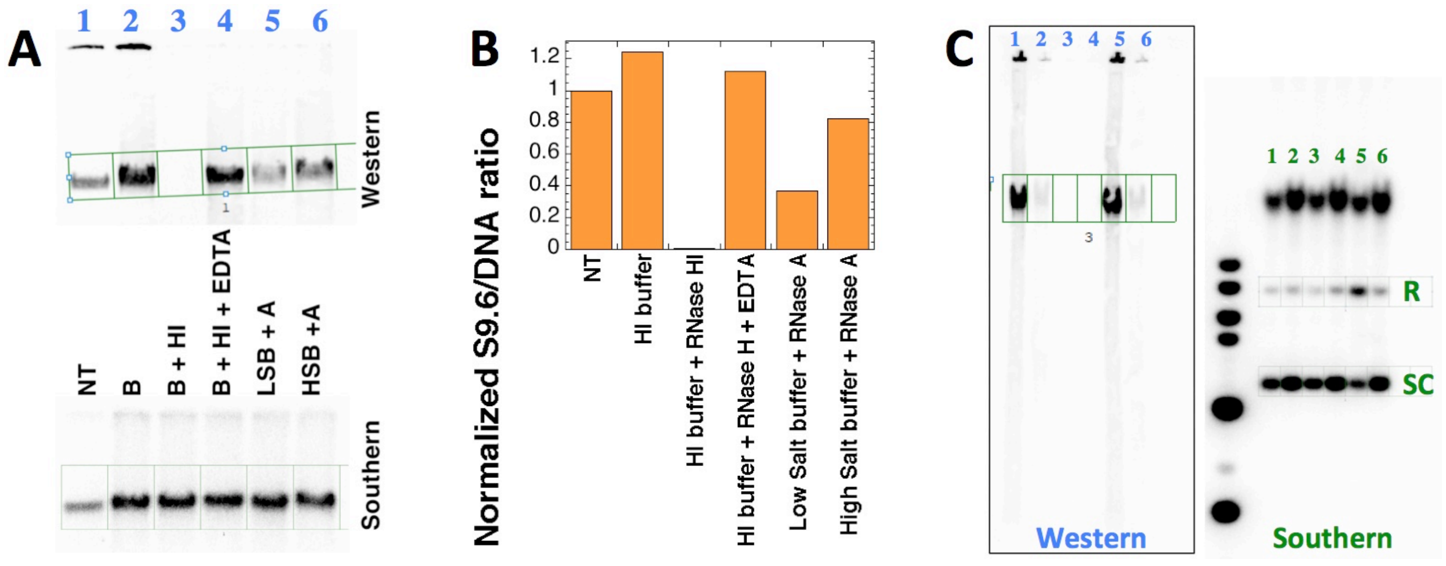

Fig. S8. Sensitivity of RDH signal in the genomic DNA to RNase treatments.

A. The RDH signal is sensitive to RNase HI treatment and to RNase A in low salt buffer (LSB + A), but resistant to RNase A in high salt buffer (HSB + A). A representative pair of a Western and Southern is shown.

B. Quantification of the gel in "A".

C. RDHs are sensitive to RNase HI, but insensitive to RNase HII. Indicated strains harboring a plasmid (DNA-rN substrate as a positive control for RNase HII activity) were irradiated with 36 J/m2 UV and incubated post-UV for one hour in the growth medium. The genomic DNA samples were loaded in two groups and after running in 0.8% agarose, were transferred to hybridization membrane. The membrane was cut in half, and the left half was hybridized with S9.6 antibodies (Western), while the right half was hybridized with the chromosomal and plasmid-specific probes (Southern), to detect plasmid relaxation by RNase HII (compare lane 1 vs 5). Loading: lanes 1, 3 and 5 — *rnhAB* (L-416) pEAK86; lanes 2, 4 and 6 —WT (AB1157) pEAK86. Treatment: lanes 1 and 2 — buffer only; lanes 3 and 4 — RNase HI; lanes 5 and 6 — RNase HII.

Second, if UV-induced S9.6 signal is indeed generated by stalled transcription, then blocking transcription initiation with rifampicin just before UV exposure should reduce the S9.6 signal. We measured S9.6 signal in the *rnhAB* strain pretreated with 100 μg/ml rifampicin for 5 min before applying 36 J/m^2 UV dose. No rifampicin was present during post-UV incubation. Nevertheless, at 60 min post-UV recovery the RNA/DNA hybrid density was reduced 2 fold (Fig. S9AB).

Third, we addressed the question of whether UV-induced S9.6 signal is dispersed all over the chromosome or confined to a few chromosomal locations, by digesting DNA from the *rnhAB uvrA* strain at 60 min post-UV recovery with restriction enzymes. We found that the S9.6 signal distribution generally follows the digested DNA pattern with EcoRI or EcoRI+BamHI digestions (Fig. S9C and Fig. 6B), indicating dispersal of the structures all over the chromosome. At the same time, we noticed that the average size of HaeII or HaeIII-digested DNA fragments is much shorter than the average size of the corresponding S9.6 signals (Fig. 6B), suggesting that longer RNA:DNA hybrids mask multiple sites of frequent cutters.

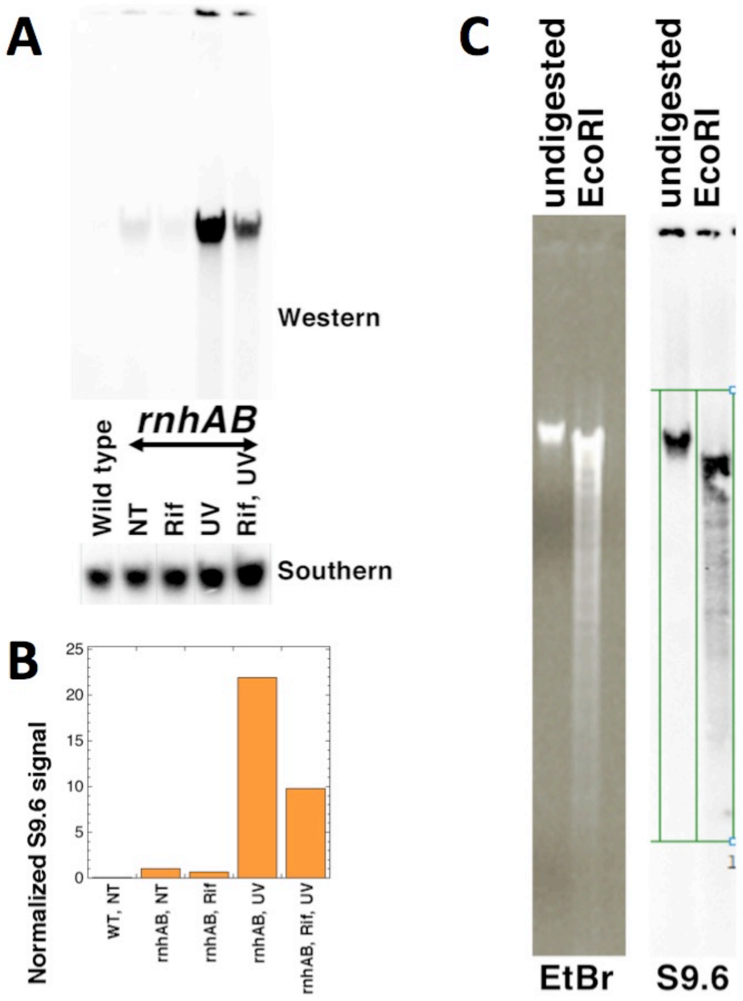

Fig. S9. Rifampicin sensitivity of the S9.6 signal and its chromosome distribution.

A. Rifampicin pretreatment reduces the UV-induced RNA:DNA hybrids. In contrast to Fig. 4E, incubation in the growth medium after UV was without rifampicin, decreasing its effect. A representative pair of a Western and Southern is shown. Strains are : WT (AB1157), *rnhAB* (L-416).

B. Quantification of the gel in "A".

C. Restriction digestion of the chromosomal DNA (L-416, 36 J/m^2 UV, one hour post-irradiation in growth medium) similarly distributes the S9.6 signal.

Fourth, we tested the thermal stability of the S9.6 signal by heating the chromosomal DNA of the *rnhAB* 60 min post-UV samples in TE buffer for one hour at 37°C, 45°C, 60°C, or at 75°C for up to 45 min, but found that both the S9.6 signal and dsDNA are equally stable (Fig. S10AB). However, the S9.6 signal is destabilized in TE buffer after 5 min at 85°, 90°or 95°C (Fig. S10C). Interestingly, at 85°C the chromosomal band is represented by the tight band of dsDNA and by a trailing smear band of denatured ssDNA, while the S9.6 signal corresponds to only dsDNA band. In other words, S9.6 signal stability depends on the duplex DNA structure, — apparently dsDNA regions around RNA:DNA hybrids are important for holding the overall structure together.

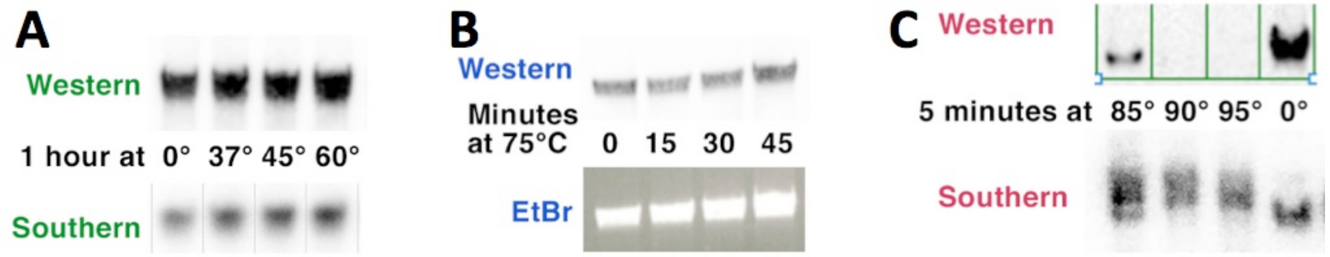

Fig. S10. The temperature stability of the RNA:DNA hybrids in the genomic DNA.

A. One hour incubation at temperatures up to 60°C.

B. Kinetics of incubation at 75°C.

C. Five minutes incubations at the indicated temperatures.

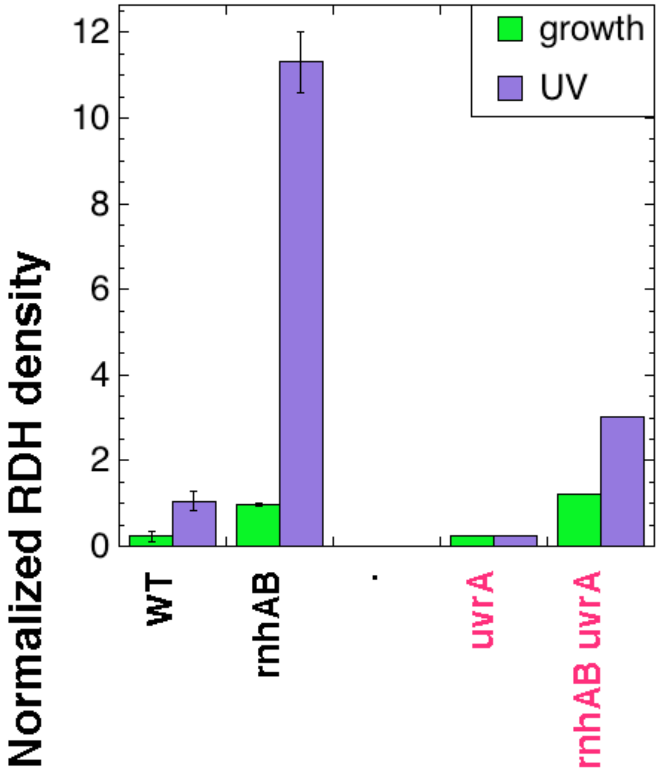

Fig. S11. The relative amount of the RNA:DNA hybrids in the chromosomal DNA of *uvrA* mutants during normal growth (green bars) versus 60 minutes after 36 J/m^2 of UV (purple bars) — as quantified in Fig. 4B. The WT and the *rnhAB* mutant results from Fig. 4B are shown for comparison. Strains; WT, AB1157; *rnhAB*, L-416; *uvrA*, SRK303; *uvrA rnhAB*, L-417.

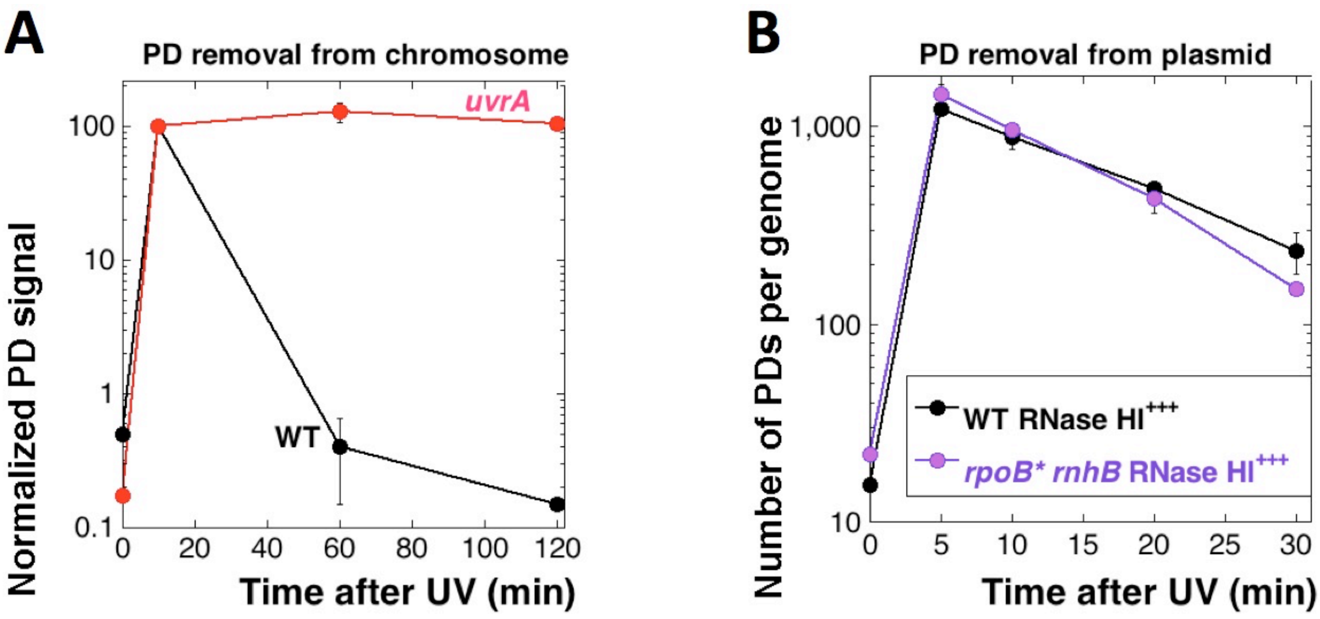

Fig. S12. PD removal from the chromosome and plasmid DNA — controls.

A. PD removal from the chromosomal DNA of the two control strains, WT (AB1157) and *uvrA* (SRK303) mutant, as quantified from gels like in "Fig. 5B". PD signal is calculated as western signal divided by the corresponding southern signal, and the value is normalized to the corresponding PD signal determined at 5 minutes (taken for 100%).

B. Kinetics of PD removal from plasmid pEAK39 in either WT strain of *rnhAB rpoB** mutant, like in Fig. 1E. The plasmid carries the *rnhA*+ gene and makes cells RNase HI+ (confirmed by genetic tests). However, this does not affect the kinetics of PD removal in WT cells (compare with Fig. 1E), while the two curves in this graph are not different by Student's statistics. Thus, we conclude that in the resulting *rnhB rpoB** strain, kinetics of PD removal is similar to the one in WT cells. Thus, NER is not accelerated in the *rpoB** (RNase HI+) mutant.

Observations about the DRIP-enrichment protocol. Sonication procedure or restriction analysis seemed not to affect the strength of the S9.6 signal when agarose gel electrophoresis was followed by the native transfer. However, we observed that short DNA fragments (both sonicated and restriction enzyme-digested) were partially lost from hybridization membrane even after increasing the dose of UV-crosslinking, — resulting in a several-fold reduction of the DNA signal. Unfortunately, the PD signal also drops in these "crushed" samples, so we were loosing PD signal disproportionally, for unknown reason. In other words, the PD signal in the HaeII/HaeIII-cut fraction is only a fraction of the PD signal of the uncut sample, due to an uncharacterized interference of the fragment size (?) with immunodetection of PDs.

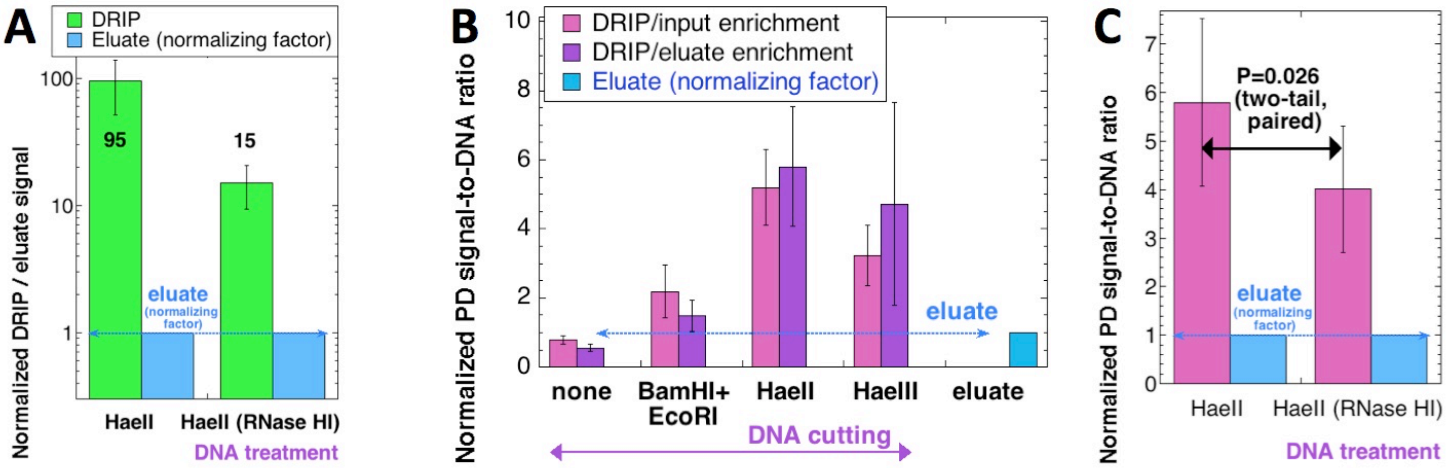

Fig. S13. Validation of the enrichment by DRIP, with normalization to the corresponding eluate (thus, eluate = 1)**.**

A. The RNase HI treatment control for the RDH enrichment by DRIP, as in Fig. 6D. Note the logarithmic scale of Y-axis.

B. The density of PDs like in Fig. 6E, but normalized to the corresponding eluate PD density, instead of the input PD density. The standard DRIP/input enrichment from Fig. 6E is shown for comparison.

C. The RNase HI treatment control for the HaeII enrichment in panel "B". Only paired data were used in this case, in contrast to Fig. 6E.

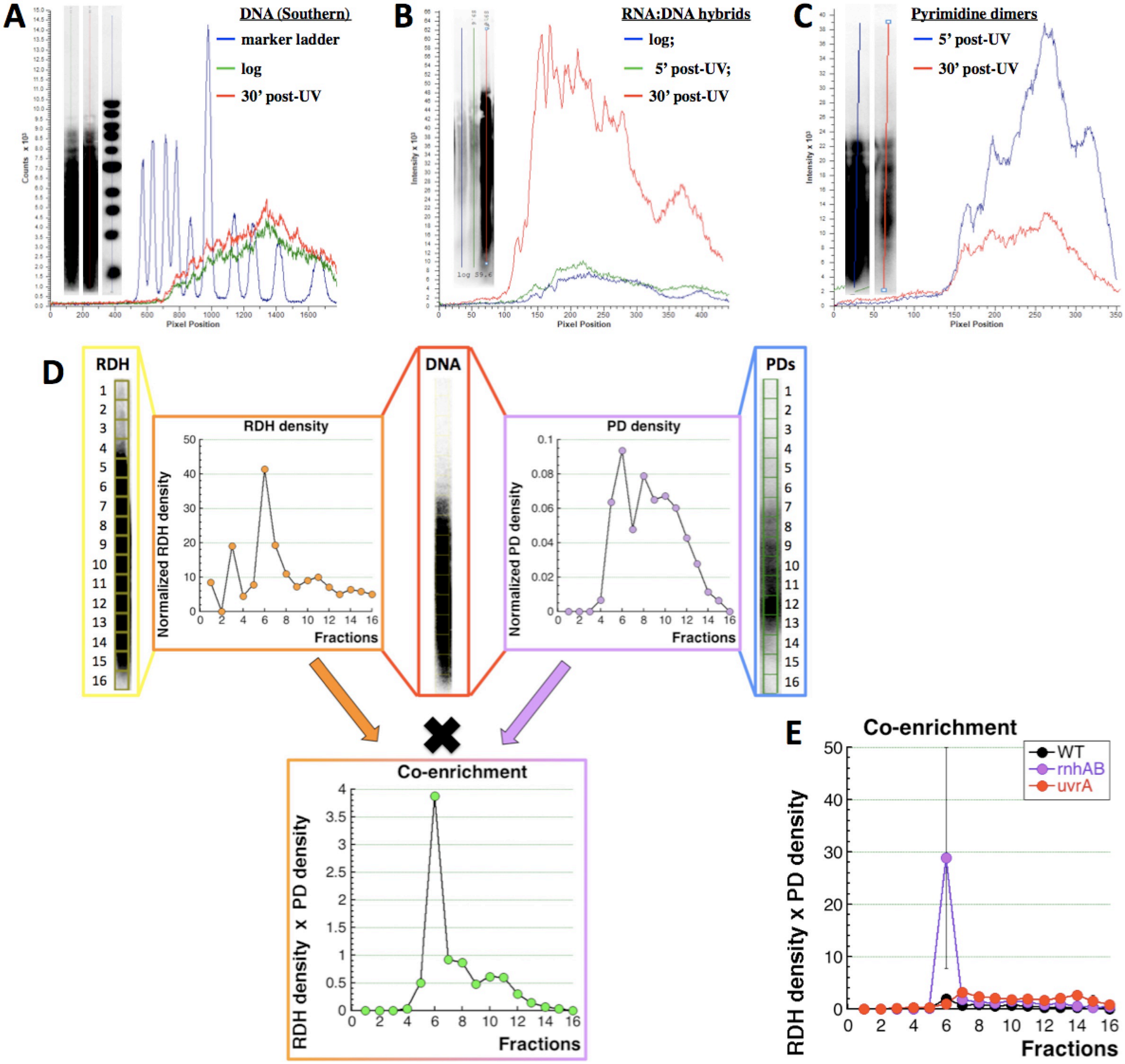

Fig. S14. The actual gel lanes and their signal profiles for the co-enrichment analysis, as well as its scheme. Samples are L-416 from a representative run.

A. A Southern of genomic DNA digested with HaeII. The 0.5 - 10.0 kb ladder is also shown. Note that the control signal (log culture) and the 30 min post-UV signal are virtually the same.

B. A Western with S9.6 antibodies for RNA:DNA hybrids. Note that the 30 min post-UV signal is much higher than the control ones (log or 5 min post-UV).

C. A Western with KTM53 antibodies for pyrimidine dimers. Note that the 30 min post-UV signal is much lower than the 5 min post-UV signal, due to the rapid repair of UV lesions in the excision repair-proficient cells.

D. A scheme of the analysis itself (described in more detail in Methods). Top: first, the normalized RDH density and, separately, normalized PD density, are quantified for a sample of interest, by dividing fractions of the Western signal by the corresponding fraction of the Southern signal of the same lane and by normalizing them, again fraction-by-fraction, to the corresponding densities at the control points (log-culture for RDH, 5 min after UV for PDs). Bottom: second, the two normalized densities are multiplied, fraction-by-fraction, to yield the co-enrichment profile of the lane.

E. Co-enrichment profile of the *rnhAB* mutant compared with ones for the WT cells and the *uvrA* mutant. (The same plot as in Fig. 7A, but with linear Y-axis to emphasize the peak differences).

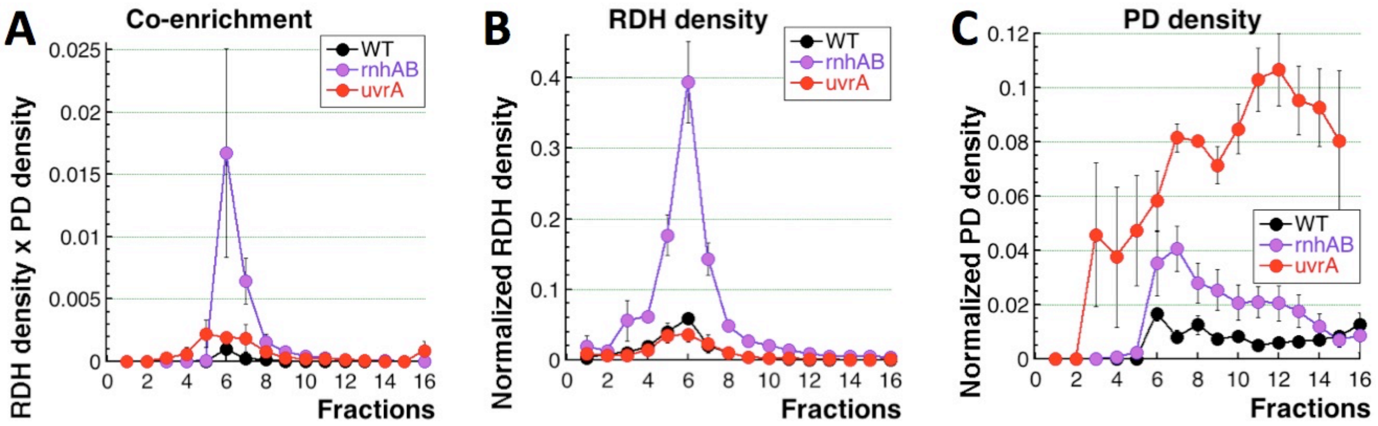

Fig. S15. Co-enrichment by lane profiling for RDH and PD signals — an alternative normalization (described below*). Compare to Fig. 7ABC, but note much smaller scales and also linear Y-axis in A (like in S14E).

A. Co-enrichment profile of the *rnhAB* mutant compared with ones for the WT cells and the *uvrA* mutant.
B. Comparison of the RDH density profiles.
C. Comparisons of the PD density profiles.

*** Enrichment calculation with the alternative normalization.**
Like in the standard protocol, the signal in the lane is partitioned into 16 equal fractions between the well and the 02.-0.5 kbp bottom of the lane, and then the density for RDH and PD are calculated for each fraction by dividing the Western signal by the Southern signal (Density = Signal PD or RDH / Signal DNA).

However, subsequent normalization of the density in individual fractions is done to the total density in the lane of the same sample: (Normalized Density RDH = Density RDH 30 min / Total Density RDH 30 min in the lane) and (Normalized Density PD = Density PD 30 min / Total Density PD30 min in the lane).

In this case, the normalized density represents the density profile of the lane. The data from individual experiments can still be averaged within the same strain, but one strain is not directly comparable to another one without consideration of their total densities first. To take into account the density differences between the three strains, we used the ratios derived from the prior dot blot analysis of the RDH and PD densities:
For RDH densities, these ratios are: L-416 / WT / uvrA = 1.00 / 0.18 / 0.15
For PD densities, these factors are: uvrA / L-416 / WT = 1.0 / 0.2 / 0.1

Thus, this calculation approach employs an external factor derived from different type of measurements, rather than direct calculation using the data within the same experiment. The assumption here is that the ratiometric factor applies to all fractions within the lane evenly.

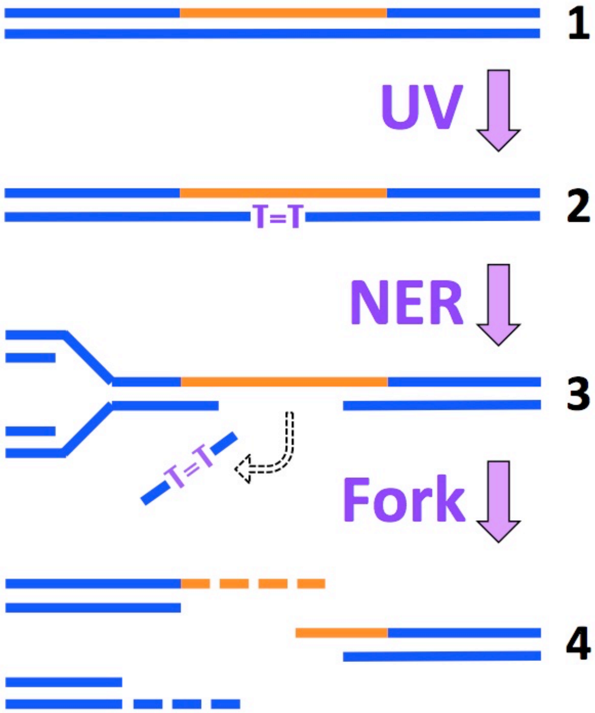

Fig. S16. This is a counterpart of Fig. 2E, but leading to an irreparable chromosome lesion. If PD across an R-tract (2) is excised by NER (3), the resulting ssRNA gap cannot be closed by synthesis and will cause replication forks explosion, via fork collapse at ss-interruption first and then degradation of the ssRNA arm. An exploded replication fork is an irreparable chromosomal lesion.

Supplemental Tables

Table S1. Strains used in this study.

Strain	Relevant genotype*	Reference
**Published**		
AB1157	Wild-type strain*	(*3*)
AM3	*ΔrecF20*::*cat*	(*4*)
ER131	*ΔrnhA*::*cat*	(*5*)
GC4803 (BH290)**	*tagA x*::*kan alkA y*::*tet*	(*6*)
JB1	*ΔrecBCD3*::*kan*	(*7*)
JDW5003/NC2943**	*dnaC2 thr*::Tn*10*	Jue D. Wang
JJC754	*ΔruvABC*::*cam*	*(8)*
JW0178-1**	*ΔrnhB782*::*kan*	CGSC #8427
JW0221-1**	*ΔdinB749*::*kan*	CGSC #8456
JW3148-1**	*greA788*::*kan*	CGSC # 10392
JW3369-1**	*greB740*::*kan*	CGSC # 10511
L-404	*ΔrnhB782*::*kan*	*(5)*
L-405	*ΔrnhB782*::*kan uvrA277*:: Tn*10*	*(5)*
L-407	*ΔrnhA*::*cat ΔrnhB782*::*kan uvrA277*:: Tn*10*	(*5*)
L-413	*ΔrnhA*	*(5)*
L-414	*ΔrnhA uvrA277*::Tn*10*	*(5)*
L-415	*ΔrnhB*	*(5)*
L-416	*ΔrnhA ΔrnhB*	(*5*)
L-417	*ΔrnhA ΔrnhB uvrA277*::Tn*10*	*(5)*
L-418	*ΔrnhB782*::*kan ΔrnhA*::*cat*	*(5)*
L-419	*ΔrnhA ΔrnhB*	*(5)*
L-431	*recF20*::*cat ΔrnhA*	(*5*)
L-435	*recF20*::*cat ΔrnhA ΔrnhB782*::*kan*	(*5*)
L-476	*recB270*(Ts) *recC271*(Ts) *ΔrnhA::cat ΔrnhB782*::*kan*	(*5*)
L-504	*dnaC2 thr*::Tn*10 ΔrnhA*::*cat ΔrnhB782*::*kan*	*(5)*
MDS42**	MG1655, 14% genome deleted	(*9*)
MG1655**	Wild-type strain	(*10*)
N3055**	*uvrA277*::Tn*10*	CGSC #6661
RSW712	MDS42 *rpoB*35 argE*::Tn*10*	(*11*)
RSW738	*Δmfd*::*kan*	(*11*)
RW82	*umuCD595*::*cat*	CGSC #7280
SK129	*recB270*(Ts) *recC271*(Ts)	(*12*)
SRK303	*uvrA277*::Tn*10*	(*5*)
**This study**		
L-301-1	*ΔgreA*	AB1157 x P1 JW3148-1, pCP20
L-306-1	*ΔgreA greB740*::*kan*	L-301-1x P1 JW3369-1
L-389	*ΔrecBCD3*::*kan*	MG1655 x P1 JB1
L-393	*dnaC2 thr*::Tn*10*	AB1157 x P1 JDW5003
L-416-33	*ΔrnhA ΔrnhB rpoB*35 argE*::Tn*10*	L-416 x P1 RSW712
L-420	*umuCD595*::*cat*	AB1157 x P1 RW82

L-423	*ΔrnhAB umuCD595*::*cat*	L-416 x P1 RW82
L-443	*umuCD595*::*cat*, *ΔdinB749*::*kan*	L-420 x P1 JW0221-1
L-446	*umuCD595*::*cat*, *ΔdinB749*::*kan ΔrnhAB*	L-423 x P1 JW0221-1
L-459	*ΔrnhB782*::*kan ΔrnhA*::*cat*	MDS42 x P1 L-418
L-462	*ΔrnhB782*::*kan ΔrnhA*::*cat rpoB*35 argE*::Tn*10*	RSW712 x P1 L-418
L-474	*ΔgreA ΔgreB*	L-306-1 pCP20
L-477	*ΔgreA ΔgreB ΔrnhB782*::*kan*	L-474 x P1 JW0178-1
L-478	*ΔgreA ΔgreB ΔrnhB782*::*kan ΔrnhA*::*cat*	L-474 x P1 L-418
L-479	*ΔgreA ΔgreB ΔrnhA*::*cat*	L-474 x P1 L-418
L-497	*ΔrnhAB umuCD595*::*cat ΔdinB*	L-446 pCP20
L-500	*umuCD595*::*cat ΔdinB*	L-443 pCP20
L-505	*Δmfd*::*kan*	AB1157 x P1 RSW738
L-506	*Δmfd*::*kan ΔrnhA*	L-413 x P1 RSW738
L-507	*Δmfd*::*kan ΔrnhB*	L-415 x P1 RSW738
L-508	*Δmfd*::*kan ΔrnhAΔrnhB*	L-416 x P1 RSW738

* — complete genotype of AB1157 includes: F– λ– rac- *thi-1 hisG4 Δ(gpt-proA)62 argE3 thr-1 leuB6 kdgK51 rfbD1 araC14 lacY1 galK2 xylA5 mtl-1 tsx-33 glnV44 rpsL31*
** — non-AB1157 background.

Table S2. Plasmids used in this study.

Plasmid	Replicon/drug resistance/other genes	Reference/derivation
**Published**		
pCY566	pMTL23/ *bla* / *cos*	*(13)*
pAM34	pAM34 (IPTG)/ *bla* / *aadA* / *lacI*	(*14*)
pEAK39	pSC101/ *aadA* / *rnhA*	(*5*)
pEAK86	pSC101*/ *bla* / *csdA*	*(5)*
pSRK1*RecG*	pBluescript/ *bla* /plac::*recG*	*(5)*
**This study**		
pSRK10-1	pLAC22/ *bla* /plac::*uvrA*	Sharik Khan (this laboratory)

Table S3. Primers.

rpoB — for sequencing *rpoB*35* mutation:

#273 TGCGTACGATCTGGGCGCTG
#274 TTACTCGTCTTCCAGTTCGATG

***ΔgreA* —** to verify deletion

#238	GTTCGTTGATAAAAGGCCG
#239	CAGGCCGAACAGCCGGGGTG

ΔgreB — to verify deletion

#240	GCTCACGTTCGTCACCAGC
#241	AGCCATCGGCAGGAGG

ΔdinB — to verify deletion

dinB-F	CGCGAATTCCGCAGCGAACGCGTTAAATG
dinB-B	AACGCTTCGAATGCGCTGGC

ΔumuCD — to verify deletion

umuCD-F	CGCGAATTCCAGTCATAATCATTCGCCTC
umuCD-B	GATCTGTTCGGTCGCTAATC

References

1. D. D. Phillips, D. N. Garboczi, K. Singh, Z. Hu, S. H. Leppla, C. E. Leysath, The sub-nanomolar binding of DNA–RNA hybrids by the single-chain Fv fragment of antibody S9.6. *J. Mol. Recogn.* **26**, 376-381 (2013).
2. S. R. Hartono, A. Malapert, P. Legros, P. Bernard, F. Chedin, V. Vanoosthuyse, The affinity of the S9.6 antibody for double-stranded RNAs impacts the accurate mapping of R-Loops in fission yeast *J. Mol. Biol.* **430**, 272-284 (2018).
3. B. J. Bachmann, in *Escherichia coli and Salmonella typhimurium. Cellular and Molecular Biology,* F. C. Neidhardt, Ed. (American Society for Microbiology, Washington, D.C., 1987), pp. 1190-1219.
4. A. Miranda, A. Kuzminov, Chromosomal lesion suppression and removal in *Escherichia coli* via linear DNA degradation. *Genetics* **163**, 1255-1271 (2003).
5. E. A. Kouzminova, F. F. Kadyrov, A. Kuzminov, RNase HII Saves *rnhA* Mutant *Escherichia coli* from R-Loop-Associated Chromosomal Fragmentation. *J. Mol. BIol.* **429**, 2873-2894 (2017)10.1016/j.jmb.2017.08.004).
6. M. Saparbaev, J. Laval, Excision of hypoxanthine from DNA containing dIMP residues by the *Escherichia coli*, yeast, rat, and human alkylpurine DNA glycosylases. *Proc. Natl. Acad. Sci. USA* **91**, 5873-5877 (1994).
7. J. S. Bradshaw, A. Kuzminov, RdgB acts to avoid chromosome fragmentation in *Escherichia coli*. *Mol. Microbiol.* **48**, 1711-1725 (2003).
8. M. Seigneur, V. Bidnenko, S. D. Ehrlich, B. Michel, RuvAB acts at arrested replication forks. *Cell* **95**, 419-430 (1998).
9. G. Pósfai, G. r. Plunkett, T. Fehér, D. Frisch, G. M. Keil, K. Umenhoffer, V. Kolisnychenko, B. Stahl, S. S. Sharma, M. de Arruda, V. Burland, S. W. Harcum, F. R. Blattner, Emergent Properties of Reduced-Genome *Escherichia Coli Science* **312**, 1044-1046 (2006).
10. F. R. Blattner, G. Plunkett III, C. A. Bloch, N. T. Perna, V. Burland, M. Riley, J. Collado-Vides, J. D. Glasner, C. K. Rode, G. F. Mayhew, J. Gregor, N. W. Davis, H. A. Kirkpatrick, M. A. Goeden, D. J. Rose, B. Mau, Y. Shao, The complete genome sequence of *Escherichia coli* K-12. *Science* **277**, 1453-1462 (1997).
11. R. S. Washburn, M. E. Gottesman, Transcription termination maintains chromosome integrity. *Proc. Natl. Acad. Sci. U.S.A.* **108**, 792-797 (2011).
12. S. R. Kushner, In vivo studies of temperature-sensitive *recB* and *recC* mutants. *J. Bacteriol.* **120**, 1213-1218 (1974).
13. J. E. Cronan, Cosmid-based system for transient expression and absolute off-to-on transcriptional control of *Escherichia coli* genes. *J. Bacteriol.* **185**, 6522-6529 (2003).
14. D. Gil, J.-P. Bouché, ColE1-type vectors with fully repressible replication. *Gene* **105**, 17-22 (1991).
